# Targeting capacity, safety and efficacy of engineered extracellular vesicles delivered by transdermal microneedles to treat plasmacytoma in mice

**DOI:** 10.1002/ctm2.70327

**Published:** 2025-05-02

**Authors:** Yulin Cao, Xuan Hu, Di Wu, Yuxuan Jiang, Yali Yu, Shan Wang, Wenlan Chen, Yaoying Long, Liuyue Xu, Jiao Qu, Bianlei Yang, Blal Chakhabi, Hongxiang Wang, Yong Deng, Lei Chen, Zhichao Chen, Qiubai Li

**Affiliations:** ^1^ Department of Rheumatology and Immunology Union Hospital, Tongji Medical College, Huazhong University of Science and Technology Wuhan China; ^2^ Cancer Center, Union Hospital, Tongji Medical College, Huazhong University of Science and Technology Wuhan China; ^3^ Department of Hematology Union Hospital, Tongji Medical College, Huazhong University of Science and Technology Wuhan China; ^4^ Britton Chance Center for Biomedical Photonics, Wuhan National Laboratory for Optoelectronics Huazhong University of Science and Technology Wuhan China; ^5^ MoE Key Laboratory for Biomedical Photonics, Department of Biomedical Engineering Huazhong University of Science and Technology Wuhan China; ^6^ Department of Hematology The Central Hospital of Wuhan, Tongji Medical College, Huazhong University of Science and Technology Wuhan China; ^7^ Hubei Engineering Research Center for Application of Extracellular Vesicle Hubei University of Science and Technology Xianning China

**Keywords:** CD38 peptide, drug delivery system, extracellular vesicles, intravenous administration, microneedles, plasmacytoma

## Abstract

**Background:**

Engineered extracellular vesicles (EVs) are emerging as a highly potential platform for targeted drug delivery in cancer therapy. Although intravenous injection is commonly used in EV treatment, there is growing interest in using microneedles (MNs) for transdermal EV delivery; however, comprehensive studies comparing the tissue distribution, safety and antitumour efficacy of these two approaches for delivering engineered EVs remain scarce.

**Methods:**

We used EVs derived from umbilical cord mesenchymal stem cells, modified with phospholipid‒polyethylene glycol‒N‐hydroxysuccinimide and conjugated with CD38 peptides (CD38‐EVs), to target myeloma cells that highly express CD38 antigen, and tested their safety and antitumour efficacy in mice with subcutaneous plasmacytoma, administrated via dissolvable transdermal MNs or intravenous injection. Flow cytometry, immunofluorescence and fluorescence molecular projection imaging analysis were employed to evaluate the distribution of CD38‐EVs at the cellular level and within living systems. Additionally, histopathological analysis and biochemical analyses were conducted to assess the antitumour effects and safety of CD38‐EVs loaded with doxorubicin (CD38‐EVs‐Dox).

**Results:**

Compared to standard EVs, CD38‐EVs exhibited enhanced uptake by CD38^high^ tumour cells and reduced uptake by CD38‐negative non‐tumour cells in vitro. In plasmacytoma NOD/SCID mouse models, CD38‐EVs encapsulated within MNs (CD38‐EVs^MNs^) effectively targeted the tumour cells much more than the standard EVs encapsulated within MNs (EVs^MNs^) and CD38‐EVs intravenously administrated (CD38‐EVs^i.v^), with reduced distribution to the lungs and spleen. Additionally, CD38‐EVs‐Dox induced significantly greater cytotoxicity against the tumour cells than EVs‐Dox in vitro, and CD38‐EVs‐Dox^MNs^ significantly reduced tumour burden compared to both EVs‐Dox^MNs^ and CD38‐EVs‐Dox^i.v^, while maintaining favourable safety profiles.

**Conclusions:**

CD38‐EVs‐Dox^MNs^ have superior efficacy and safety in treating plasmacytoma mice, compared to CD38‐EVs‐Dox^i.v^, providing novel insights into the potential of MNs as a platform for delivering targeted engineered EVs in tumour therapy.

**Highlights:**

- **Enhanced tumor targeting**: CD38‐modified EVs (CD38‐EVs) showed increased uptake by CD38high tumor cells while reducing uptake by CD38‐negative non‐tumor cells.
- **Optimized delivery**: MN‐loaded CD38‐EVs targeted tumors more effectively than MN‐loaded EVs and intravenously injected CD38‐EVs, with lower lung and spleen accumulation.
- **Superior antitumor efficacy**: MN‐delivered CD38‐EVs‐Dox significantly suppressed tumor growth, outperforming intravenous CD38‐EVs‐Dox and MN‐delivered EVs‐Dox.

## Graphical abstract

A schematic illustration of engineered EVs (CD38‐EVs‐Dox) delivered via MNs or intravenous administration for plasmacytoma treatment. CD38‐EVs encapsulated within MNs effectively enhanced tumour cell targeting compared to standard EVs in MNs and intravenously administered CD38‐EVs, with reduced lung and spleen distribution.

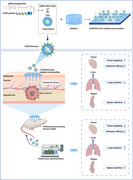

## 1 INTRODUCTION

Drug delivery systems targeting neoplastic tissues are gaining interest due to their potential to enhance therapeutic effects and minimise treatment‐induced damage.^1^ Extracellular vesicles (EVs), such as exosomes and microvesicles, are garnering attention as nanoscale drug carriers,^2^ offering advantages over conventional nanocarriers, including low immunogenicity, excellent biocompatibility, the capacity to cross the biological barrier, engineering potential, and efficient cargo delivery.^3^ These characteristics position EVs as highly promising candidates for targeted drug delivery in tumour treatment.

EVs used for therapeutic delivery are typically isolated from a variety of sources, including mesenchymal stem cells (MSCs), tumour cells, blood and engineered cell lines such as HEK293T. Among these, MSC‐derived EVs exhibit several advantages, including low immunogenicity, non‐tumourigenicity and broad tissue availability, making them well‐suited for clinical translation.^4^ In contrast, tumour cell‐derived EVs may carry oncogenic cargo, potentially promoting malignancy, while HEK293T‐derived EVs may contain proliferative signals or oncogenic miRNAs, raising safety concerns.^5^, ^6^ Although blood‐derived EVs are abundant, their heterogeneity—resulting from contaminants such as apoptotic bodies and immune cell remnants—limits their clinical applicability.^7^ Recently, umbilical cord‐derived MSCs have gained attention as an optimal EV source due to their high proliferative capacity and favourable ethical profile, highlighting their potential for therapeutic applications.^8^

Despite these advantages, the progression of EV‐based therapeutics into clinical use is hindered by challenges related to delivery efficiency, biodistribution and targeting specificity. EVs can currently be delivered orally, intravenously, through local injection, or via aerosol inhalation. Intravenous (i.v) injection is commonly employed in both preclinical and clinical research, particularly in tumour therapy.^9^, ^10^, ^11^ However, EVs predominantly accumulate in the liver and spleen rather than in neoplastic tissues, especially for tumours located away from vascular‐rich regions.^12^ The biodistribution of EVs varies depending on the application method.^13^ Assessing the systemic organ biodistribution and tumour‐targeting efficiency of EVs is essential for refining delivery strategies.

Concurrently, there is growing interest in using EV‐loaded microneedles (MNs) for transdermal drug delivery.^14^ These portable, minimally invasive devices create temporary microchannels in the skin, facilitating drug entry into the bloodstream and lymphatic system, thereby treating conditions such as infectious diseases, diabetes and cancer.^15^, ^16^ Recent studies indicate that MN‐based delivery can circumvent first‐pass hepatic metabolism and mitigate systemic toxicity while facilitating the sustained release of therapeutic cargo.^17^, ^18^ However, there is a lack of comprehensive research comparing tissue distribution, targeting efficiency, biological safety and antitumour efficacy of engineered EVs delivered via i.v administration and MNs methods.

Multiple myeloma (MM) is the second most common haematologic malignancy, characterised by the neoplastic clonal expansion of plasma cells in the bone marrow.^19^ Despite advancements, current treatments such as immunomodulatory agents and proteasome inhibitors are frequently hindered by drug resistance and severe off‐target effects.^20^ CD38, the target of the FDA‐approved monoclonal antibody daratumumab, serve as a promising receptor for assessing targeted drug delivery strategies.^21^, ^22^ To improve the targeting capability of native EVs, surface engineering strategies such as peptide conjugation, antibody modification and receptor‒ligand coupling have been explored.^23^, ^24^ Among these, peptide engineering offers a cost‐effective and scalable method to improve tumour‐specific homing while preserving EV stability.^25^ Several studies have been conducted on CD38‐targeted nanoparticles loaded with chemotherapeutic agents for MM treatment^26^, ^27^, ^28^, ^29^; however, no studies of engineered CD38‐targeted EVs within MNs have been reported.

In this study, we used plasmacytoma‐bearing NOD/SCID mice as tumour models and developed CD38 peptide‐engineered EVs (CD38‐EVs) from umbilical cord MSCs to target malignant plasma cells. We compared the effectiveness of these EVs in targeting tumour cells in the mice between i.v tail administration and transdermal MN encapsulation and assessed the organ distribution, antitumour efficacy and safety of doxorubicin‐loaded CD38‐EVs (CD38‐EVs‐Dox) using these two methods, aiming to innovate delivery strategies for engineered EVs in targeted therapy of plasmacytoma.

## 2 RESULTS

### 2.1 Construction and characterisation of CD38‐EVs

**FIGURE 1:**
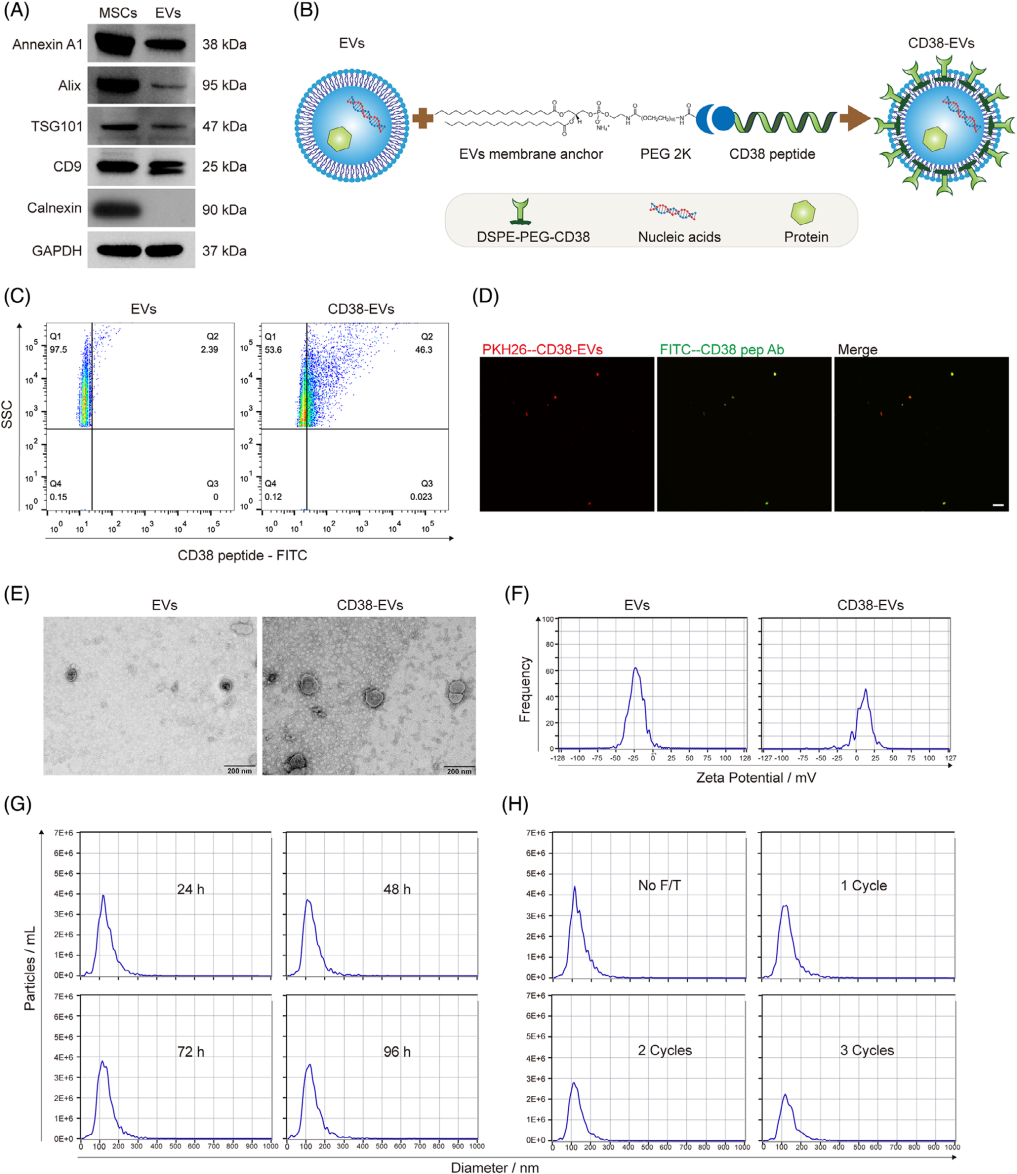
Generation and characterisation of CD38‐EVs. (A) Western blot analysis of extracellular vesicle (EV) marker proteins (Annexin A1, Alix, TSG101, CD9) and negative marker (Calnexin) in mesenchymal stem cells (MSCs) and EVs. (B) Schematic representation of the illustration of the CD38‐EVs construction process. (C) Nano flow cytometry (FCM) analysis of the CD38pep surface marker of EVs and CD38‐EVs using anti‐CD38pep antibody. (D) Fluorescein 5‐isothiocyanate (FITC)‐labelled anti‐CD38pep antibody (Ab) binding with CD38‐EVs observed by fluorescence microscope (scale bars: 20 µm). (E) Representative transmission electron microscopy (TEM) images of EVs and CD38‐EVs (scale bars: 200 nm). (F) Zeta potential measurements of EVs and CD38‐EVs using a ZetaView analyser. (G) Nanoparticle tracking analysis (NTA) of CD38‐EVs stored at 4°C for different durations, assessing size distribution and concentration. (H) NTA evaluation of CD38‐EVs following freezing and thawing (F/T) cycles at ‒80°C, analysing changes in size distribution and concentration.

The isolated EVs expressed characteristic markers, including Annexin A1, Alix, TSG101 and CD9, but lacked Calnexin expression (Figure 1A). EV yield was quantified based on protein concentration and particle count. The protein concentration of EVs was .27 ± .04 µg/mL, with an inter‐batch coefficient of variation (CV) of 14.79%. The particle concentration was (1.59 ± .16) × 10^9^ particles/mL, with an inter‐batch CV of 10.51% (Table S1). These results demonstrate the high purity and batch‐to‐batch consistency of the isolated EVs (CV < 15%).

We engineered CD38‐EVs (Figure 1B) to target CD38^high^ myeloma cells. After obtaining and characterising the EVs as we previously described,^4^, ^30^, ^31^ we synthesised the fluorescein 5‐isothiocyanate (FITC)‐labelled anti‐CD38 peptide (CD38pep, ARGDYYGSNSLDYW)^27^ and confirmed their binding to the CD38^high^ myeloma cells. Fluorescence overlap (yellow) was observed only in PKH26‐stained RPMI8226 and U266 (red) myeloma cells, but not in CD38‐negative MSCs or K562 leukaemia cells, demonstrating that the synthesised CD38pep specifically bound to CD38‐positive myeloma cells (Figure S1). CD38pep‒lipid conjugates (DSPE‐PEG‐CD38) were then constructed by linking CD38pep to the lipid (1,2‐distearoyl‐sn‐glycero‐3‐phosphoethanolanmine, DSPE) via a PEG2000 (approximately 45 ethylene glycol units long) linker (Figure S2a), and characterised by hydrogen nuclear magnetic resonance spectroscopy (1H‐NMR) (Figure S2b). DSPE‐PEG‐CD38 was then added to the EV‒phosphate‐buffered saline (PBS) solution (20 µg EVs protein amount: 2 µg DSPE‐PEG‐CD38 compound) and incubated at 25°C for 1 h, allowing DSPE to integrate into the EVs' lipid bilayer and form CD38‐EVs (Figure S3). To confirm the construction of engineered CD38‐EVs, we synthesised a FITC‐labelled anti‐CD38pep antibody to identify their membrane expression of CD38pep. Nano flow cytometry (FCM) analysis confirmed the expression of CD38pep in CD38‐EVs (Figure 1C). Under a fluorescence microscope, PKH26‐labelled CD38‐EVs (red) wholly merged with the anti‐CD38pep antibody (green) (Figure 1D). These results confirm the successful generation of CD38‐EVs coated with CD38pep.

We next evaluated CD38‐EVs in morphology, size, zeta potential and stability. Transmission electron microscopy (TEM) imaging showed that both EVs and CD38‐EVs exhibited a round or oval vesicular structure with an intact phospholipid bilayer. CD38‐EVs had a larger spheroidal shape due to the PEG cloud coating (Figure 1E) and a less negative surface charge (Figure 1F) compared to EVs. Nanoparticle tracking analysis (NTA) indicated that the size distribution and particle count of CD38‐EVs stayed unchanged when preserved at 4°C for up to 96 h (Figure 1G). However, repeated freezing and thawing (F/T) cycles significantly decreased particle numbers without altering their size (Figure 1H).

### 2.2 Capacity of CD38‐EVs targeting tumour cells in vitro

FCM analysis using a CD38 antibody was performed to assess CD38 expression across the studied cell lines. The results showed CD38 positivity in RPMI8226 and U266 cells, whereas human umbilical vein endothelial cells (HUVECs) and bone marrow stromal/stem cells (BMSCs) exhibited no detectable CD38 expression (Figure S4).

**FIGURE 2:**
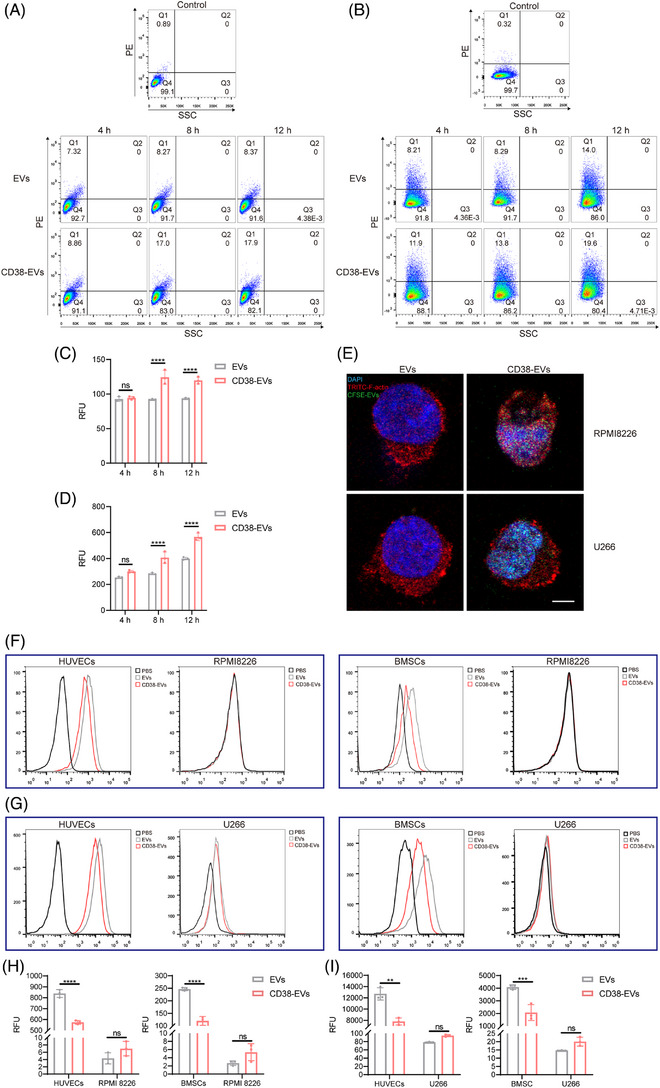
CD38‐EVs targeting tumour cells in vitro. PKH26‐labelled extracellular vesicles (EVs) and CD38‐EVs incubated with RPMI8226 cells (A) and U266 cells (B) for 4, 8 and 12 h, with cell fluorescence measured by flow cytometry (FCM). Kinetics of uptake of EVs and CD38‐EVs within 12 h by RPMI8226 cells (C) and U266 cells (D), measured by relative fluorescence units (RFU). (E) RPMI8226 and U266 cells were incubated with EVs and CD38‐EVs for 8 h and visualised using laser scanning confocal microscope (LSCM) (scale bars: 5 µm). PKH26‐labelled EVs and CD38‐EVs incubated with RPMI8226/HUVECs and RPMI8226/BMSCs (F) and U266/HUVECs and U266/BMSCs (G) co‐culture systems for 12 h, with cell fluorescence measured by FCM. Kinetics of uptake of EVs and CD38‐EVs by myeloma cells, human umbilical vein endothelial cells (HUVECs) and bone marrow stromal/stem cells (BMSCs) in RPMI8226/HUVECs and RPMI8226/BMSCs (H) and U266/HUVECs and U266/BMSCs (I) co‐culture systems, measured by RFU. Data are generated from three independent experiments.

To compare the differential uptake of CD38‐EVs and EVs by myeloma cells, PKH26‐labelled EVs and CD38‐EVs were co‐incubated with RPMI8226 and U266 myeloma cells for 4, 8 and 12 h. Uptake was measured by fluorescence intensity using FCM (Figure 2A,B). Results showed significantly higher uptake of CD38‐EVs by both RPMI8226 (Figure 2C) and U266 (Figure 2D) cells at 8 and 12 h, as confirmed by laser scanning confocal microscopy (LSCM), which showed the internalisation of CD38‐EVs into myeloma cells (Figure 2E). To determine whether CD38‐EVs interact with surface CD38 on myeloma cells, U266 cells were preincubated with a CD38‐blocking peptide before exposure to PKH26‐labelled CD38‐EVs or control EVs. Notably, CD38‐EVs did not exhibit increased targeting capability compared to EVs after CD38 antigen blockade (Figure S5). To determine CD38‐EVs binding to myeloma cells, we developed in vitro co‐culture systems with myeloma cells and either BMSCs (RPMI8226/BMSCs and U266/BMSCs) or HUVECs (RPMI8226/HUVECs and U266/HUVECs). FCM was employed to assess the uptake of EVs and CD38‐EVs by these cells, with a comparative analysis of uptake rates between tumour and non‐tumour cells. The basal uptake rates of both types of EVs by HUVECs and BMSCs were markedly higher compared to myeloma cells (Figure 2F,G). Although myeloma cells appeared to take up more CD38‐EVs than EVs, the difference was insignificant. Interestingly, CD38‐negative non‐tumour cells in the co‐culture systems (Figure 2H,I) took up significantly fewer CD38‐EVs than EVs. These findings demonstrate that CD38‐EVs are more effective than EVs at targeting myeloma cells and reducing uptake by non‐tumour cells, supporting CD38‐EVs as a promising targeted therapeutic vector.

### 2.3 Failure of intravenously administered CD38‐EVs to target tumour cells in vivo

**FIGURE 3:**
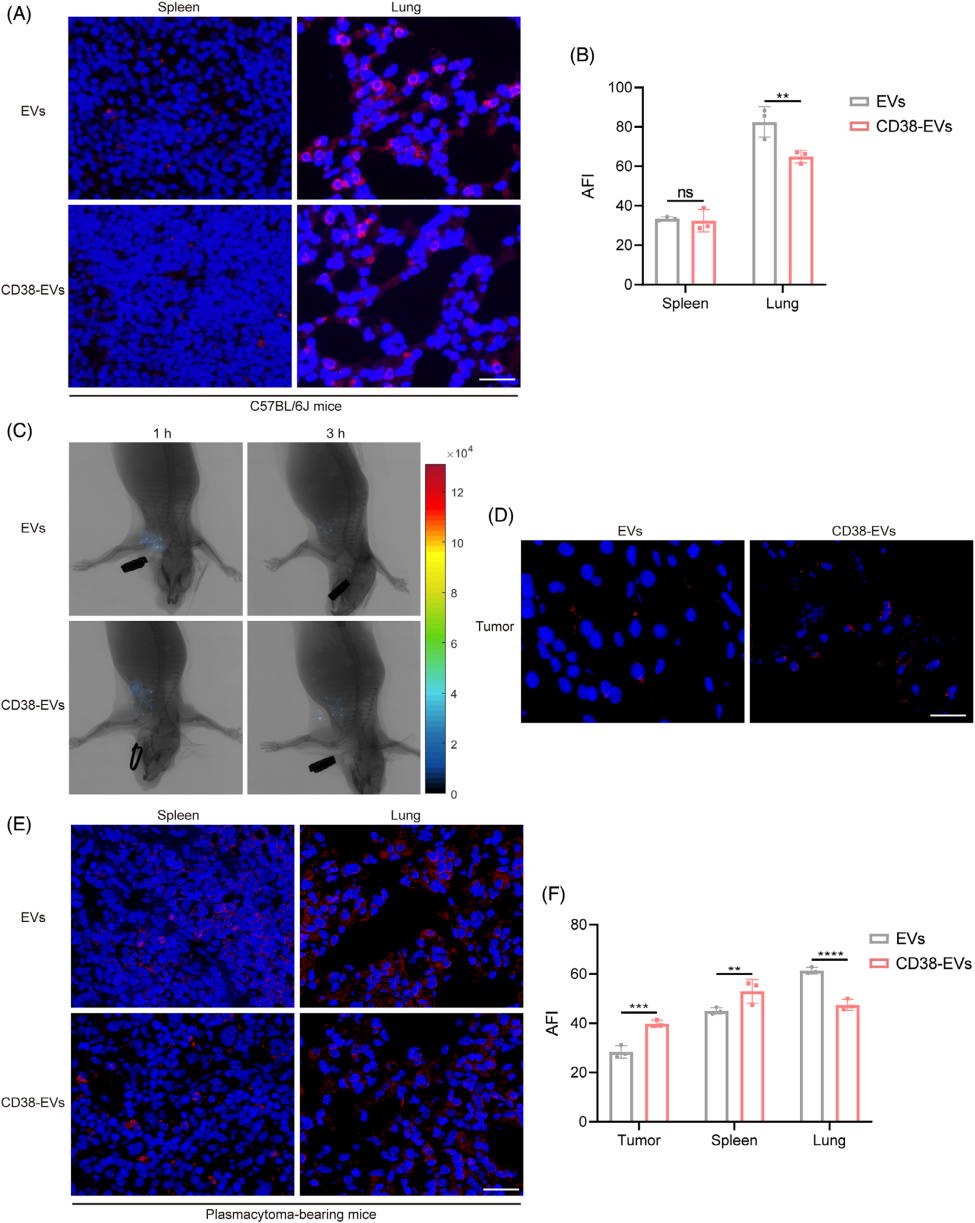
Intravenously administrated CD38‐EVs exhibit limited tumour targeting in plasmacytoma‐bearing mice. (A) Representative fluorescence microscopic images of intravenous PKH‐26‐labelled extracellular vesicles (EVs) and CD38‐EVs in spleen and lung tissues at 3 h in C57BL/6J mice (scale bars: 20 µm). (B) Measurement of the average fluorescence intensity (AFI) in spleen and lung tissue sections from panel (A) (*n* = 3). (C) In vivo representative fluorescence molecular projection (FMP) images of plasmacytoma‐bearing mice following intravenous injection of DiR‐labelled EVs and CD38‐EVs at 1 and 3 h. (D) Representative fluorescence microscopic images of intravenous PKH‐26‐labelled EVs and CD38‐EVs in tumour tissues (scale bars: 20 µm). (E) Representative fluorescence microscopic images of intravenous PKH‐26‐labelled EVs and CD38‐EVs in spleen and lung at 3 h in plasmacytoma‐bearing mice (scale bars: 20 µm). (F) Measurement of the AFI in the tumour, spleen and lung tissue sections from panels (D and E) (*n* = 3).

Unlike the fluorescent molecular imaging (FMI), our developed fluorescence molecular projection (FMP) imaging system^32^ used a precisely directed near‐infrared beam instead of diffuse wide‐field illumination, allowing for deeper penetration and lower doses to excite the fluorescent target and enhancing fluorescence molecular tomography for better in vivo imaging and analysis of EVs distribution (Figure S6). We then examined the distribution of EVs and CD38‐EVs in normal C57BL/6J mice after i.v injection. Both primarily accumulated in the lungs and spleen, with the lungs showing the highest fluorescence intensity (Figure 3A). Notably, CD38‐EVs had significantly lower lung retention than EVs, although no notable difference was detected in the spleen (Figure 3B). We then created plasmacytoma‐bearing NOD/SCID mouse models by injecting CD38‐EVs via the tail and used the FMP imaging system to observe tumour tissues in vivo and ex vivo. Although CD38‐EVs were more accessible to tumour cells at the tumour site than EVs after i.v injection, they also predominantly accumulated in the spleen and lungs rather than the tumour site (Figure 3C‒F). CD38‐EVs had significantly lower lung retention than EVs in plasmacytoma‐bearing mice, similar to normal C57BL/6J mice (Figure 3B,F). However, CD38‐EVs were notably more concentrated in the spleen of plasmacytoma‐bearing mice (Figure 3F). No significant variations in biodistribution were observed in the heart, liver, kidneys or brain between the two models (Figure S7). These findings indicate that intravenously administered CD38‐EVs exhibit greater accessibility to tumour cells compared to EVs, and significantly reduce distribution in the lungs; however, the CD38‐EVs fail to efficiently accumulate at the tumour site.

### 2.4 MN‐encapsulated CD38‐EVs effectively target tumour cells in vivo

**FIGURE 4:**
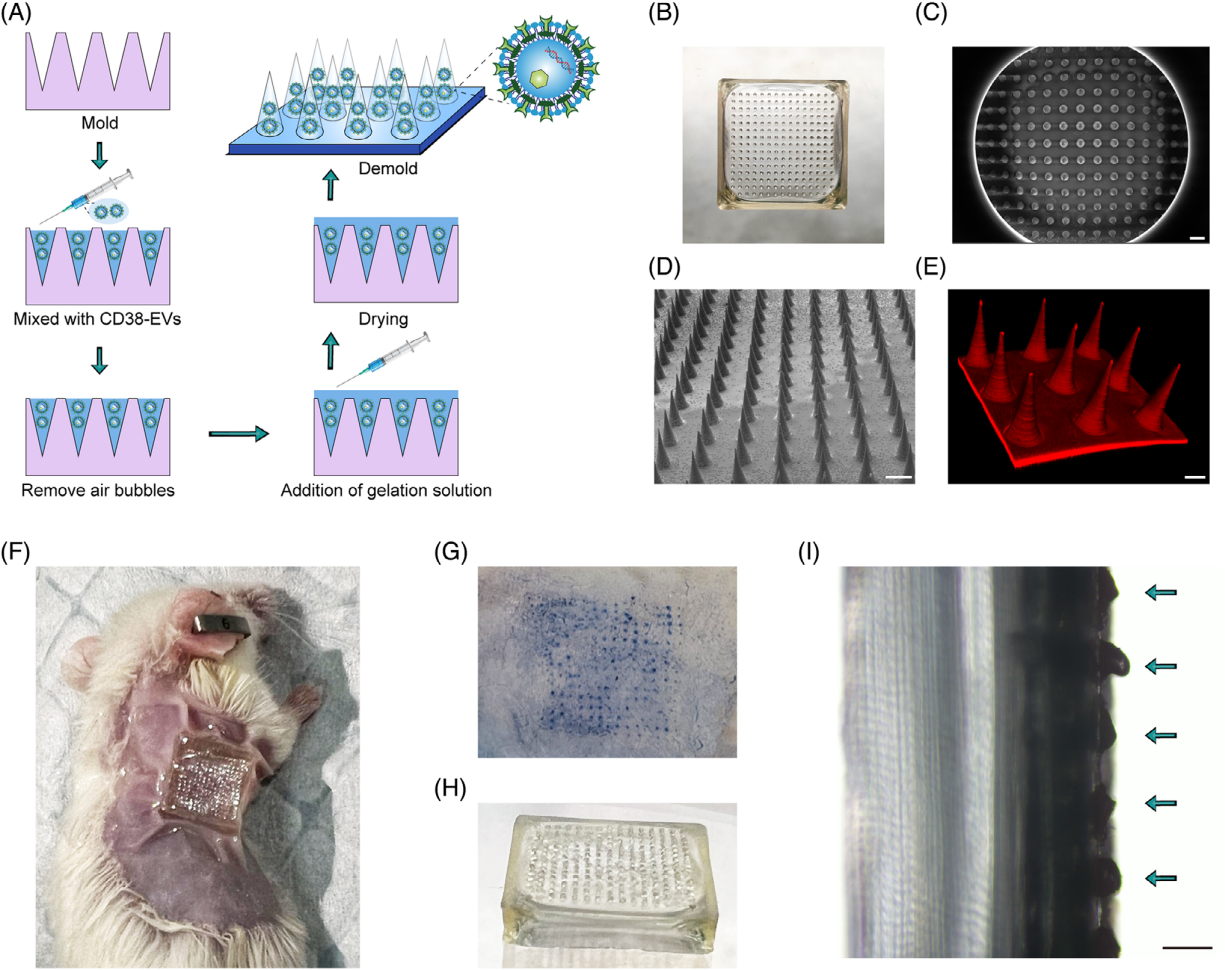
Generation and characterisation of microneedles (MNs) encapsulating CD38‐EVs. (A) Schematic illustration of fabrication of the gelation MNs embedded with CD38‐EVs. (B) Optical image of the MNs. Scanning electron microscopy (SEM) images showing the MNs from the top view (C) and side view (D) (scale bars: 500 µm). (E) Laser scanning confocal image of PKH‐26 staining MNs (scale bars: 20 µm). (F) Representative image of a plasmacytoma‐bearing mice mouse with MNs. (G) Photograph of the excised mouse skin was stained with trypan blue after MN insertion. (H) Optical image of the MNs after insertion. (I) Photographs of the micro‐camera view of MNs after MNs were inserted into the skin, the arrows indicate the dissolved cone needles (scale bars: 200 µm). EV, extracellular vesicle.

EV‐loaded MNs hold great potential for transdermal drug delivery.^14^, ^33^ To deliver CD38‐EVs directly to the tumour site in mice, we used dissolvable MNs for encapsulation. Figure 4A shows the gelatin‐based MN fabrication process with CD38‐EVs. The assembled MNs were sealed in aluminum foil pouches and preserved at 4°C for subsequent use. The MNs, sized around 11 mm × 11 mm with 225 needles, adequately covered the subcutaneous tumour site (Figure 4B). Scanning electron microscopy (SEM) images revealed the conical MNs feature a 280 µm base, 600 µm height and 10 µm tip diameter (Figure 4C,D). The MNs showed consistent needle distribution and size under SEM and LSCM (Figure 4D,E). Figure 4F shows plasmacytoma‐bearing mice treated with MNs. We tested MNs' skin penetration ability, noting distinct trypan blue spots at insertion sites (Figure 4G), confirming their mechanical strength for stratum corneum penetration. The MNs, made from biodegradable gelatin methacryloyl GelMA,^34^ dissolved upon exposure to skin interstitial fluid. Figure 4H,I shows the optical image post‐insertion and the micro‐camera view 3 h later, illustrating the dissolved cone‐shaped needles. These results confirm the successful construction of MNs encapsulated with CD38‐EVs (CD38‐EVs^MNs^).

**FIGURE 5:**
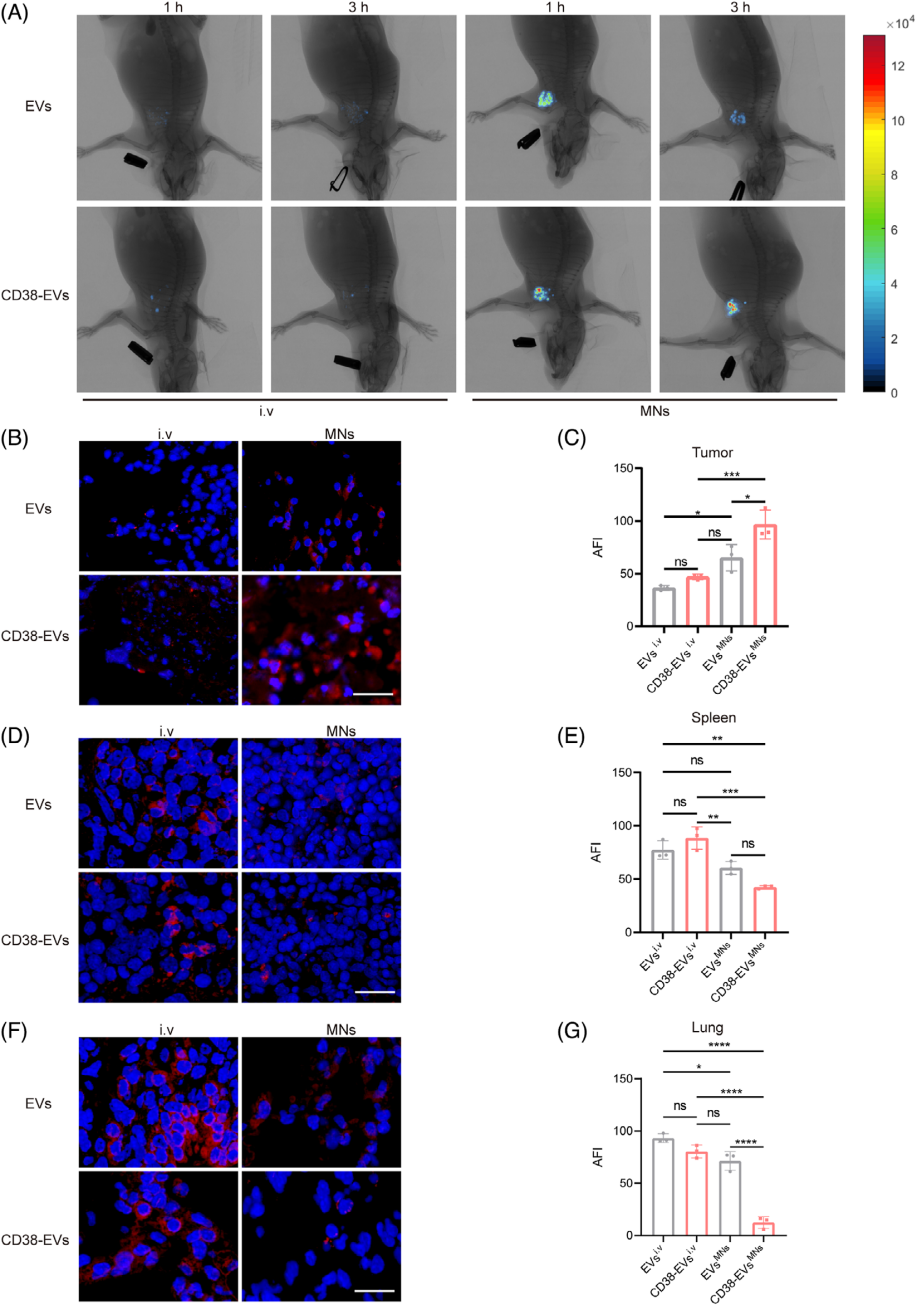
CD38‐EVs^MNs^ efficiently target tumour cells in vivo. (A) In vivo representative fluorescence molecular projection (FMP) images of plasmacytoma‐bearing mice treated with DiR‐labelled extracellular vesicles (EVs) and CD38‐EVs via intravenous (i.v) injection and microneedles (MNs) at 1 and 3 h. (B) Representative fluorescence microscopic images of the distribution of PKH‐26‐labelled EVs and CD38‐EVs in tumour tissues via i.v injection and MNs at 3 h, respectively (scale bars: 50 µm). (C) Measurement of the average fluorescence intensity (AFI) in the tumour tissue sections from panel (B) (*n* = 3). (D) Fluorescence microscopic images of the distribution of PKH‐26‐labelled EVs and CD38‐EVs in spleen tissue sections via i.v injection and MNs at 3 h (scale bars: 20 µm). (E) Measurement of the AFI in the spleen tissue sections from panel (D) (*n* = 3). (F) Fluorescence microscopic images of the distribution of PKH‐26‐labelled EVs and CD38‐EVs in lung tissue sections via i.v injection and MNs at 3 h (scale bars: 20 µm). (G) Measurement of the AFI in the lung tissue sections from panel (F) (*n* = 3).

To investigate whether CD38‐EVs^MNs^ could effectively target and reach the tumour cells in mice, four groups (*n* = 6 per group) were treated with EVs^i.v^, CD38‐EVs^i.v^, EVs^MNs^ and CD38‐EVs^MNs^, respectively. Fluorescence intensities of DiR‐labelled EVs and CD38‐EVs (*n* = 3 per group), as well as PKH‐26‐labelled EVs and CD38‐EVs (*n* = 3 per group), were measured at various times using the FMP in vivo imaging system and ex vivo tumour tissue observation, comparing the tumour‐targeting efficiency of CD38‐EVs^MNs^ with CD38‐EVs^i.v^. Notably, EVs^MNs^ and CD38‐EVs^MNs^ groups showed significantly better tumour site delivery than EVs^i.v^ and CD38‐EVs^i.v^ groups at 1 and 3 h post‐administration (Figure 5). CD38‐EVs^MNs^ exhibited the highest fluorescence intensity among all groups (Figure 5A), with fluorescence microscopy of tumour tissue sections 3 h post‐treatment further confirming the most robust PKH‐26 staining in the CD38‐EVs^MNs^ group (Figure 5B,C). Additionally, the average fluorescence intensities (AFIs) of tumour tissue in the CD38‐EVs^i.v^ and CD38‐EVs^MNs^ groups were markedly higher compared to the EVs^i.v^ and EVs^MNs^ groups, respectively, with a significant statistical difference observed between the two MN groups. Meanwhile, the two MN groups showed significantly higher AFI than the two i.v groups, respectively. Our engineering and MN delivery methods improve EV distribution and tumour cell targeting.

EVs^MNs^ and CD38‐EVs^MNs^ can enter the systemic circulation via capillaries and lymphatic vessels, reaching significant organs such as the lungs and spleen. Therefore, we measured the AFIs in these tissues across four distinct groups. Our findings revealed that the AFIs were significantly reduced in the spleen and lungs of the CD38‐EVs^MNs^ group in comparison to the EVs^MNs^ group (Figure 5D‒G), with no significant differences in other organs (heart, liver, kidneys and brain) (Figure S7c,f). These results demonstrate that EV engineering, especially administrated through MN delivery, imparts CD38‐EVs with the capability to reduce absorption by the spleen and lungs, which are typically the primary sites of EV distribution. It is noteworthy that CD38‐EVs exhibit significantly higher absorption when administrated intravenously but demonstrate reduced uptake in the spleen when delivered via MNs compared to EVs. This observation underscores the potential advantage of MNs over i.v administration.

Collectively, these findings indicate that CD38‐EVs^MNs^ can efficiently and site‐specifically target tumour cells in mice while significantly limiting their distribution in the spleen and lungs.

### 2.5 Antitumour effect of CD38‐EVs^MNs^ loaded with doxorubicin

Subsequently, we encapsulated CD38‐EVs‐Dox and evaluated their antitumour efficacy (Figure S8). The CD38‐EVs‐Dox exhibited red fluorescence, retained a spheroidal shape with a bilayer membrane, and displayed no morphological abnormalities under TEM (Figure S8b,c). The NTA and ZetaView analyser revealed that CD38‐EVs‐Dox showed a size range of 50‒300 nm and a zeta potential ranging from ‒25 to +25 mV, matching CD38‐EVs characteristics (Figure S8d,e). The Dox loading efficiency into CD38‐EVs was quantified as 21.88 ± 1.16% using liquid chromatography‒tandem mass spectrometry (LC‒MS/MS) (Figure S8f and Table S2).

**FIGURE 6:**
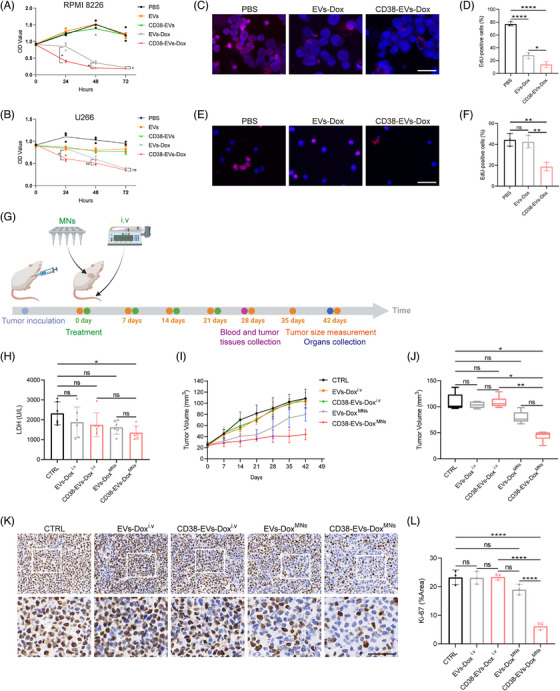
Antitumour effect of CD38‐EVs^MNs^ loaded with doxorubicin in vivo. The proliferation of RPMI8226 cells (A) and U266 cells (B) cultured in the presence of phosphate‐buffered saline (PBS), extracellular vesicles (EVs), CD38‐EVs, EVs‐Dox and CD38‐EVs‐Dox (*n* = 3). Representative 5‐ethynyl‐2′‐deoxyuridine (EdU) staining and quantification of RPMI8226 cells (C and D) and U266 cells (E and F) cultured with PBS, EVs‐Dox and CD38‐EVs‐Dox (*n* = 3) (scale bars: 50 µm). (G) Schematic illustration of the antitumour study. (H) Lactate dehydrogenase (LDH) value in the serum (*n* = 6). (I) Tumour volume changes of mice with different treatments in 42 days. (J) Statistical analysis of tumour volume in all treatment groups at 42 days. (K) Representative images of Ki‐67 immunohistochemistry (IHC) staining in tumour tissues from the different treatment groups (scale bars: 50 µm). (L) Quantitative analysis of Ki‐67 expression, presented as the ratio of positive area to total area (*n* = 3).

We incubated RPMI8226 and U266 cells with PBS, EVs, CD38‐EVs, EVs‐Dox and CD38‐EVs‐Dox. Cell viability was evaluated via cell counting kit‐8 (CCK‐8) assay, which revealed that CD38‐EVs‐Dox notably inhibited the proliferation of RPMI8226 cells at all time points, as well as U266 cell proliferation at 24 h, in comparison to EVs‐Dox (Figure 6A,B). Given that Dox inhibits DNA synthesis of tumour cells,^35^ we conducted a 5‐ethynyl‐2′‐deoxyuridine (EdU) incorporation assay on RPMI8226 (Figure 6C) and U266 (Figure 6E) cells. Red fluorescence indicates EdU‐incorporated cells and blue fluorescence marks Hoechst‐stained nuclei. The cells were incubated with PBS, EVs‐Dox and CD38‐EVs‐Dox for 24 h. The results demonstrated that CD38‐EVs‐Dox significantly inhibited DNA replication in RPMI8226 (Figure 6D) and U266 (Figure 6F) cells more effectively than EVs‐Dox. This confirms the enhanced cytotoxicity of CD38‐EVs‐Dox against tumour cells, aligning with the observed differences in cellular uptake between CD38‐EVs and EVs.

We next evaluated the in vivo antitumour efficacy of CD38‐EVs‐Dox delivered by MNs. The plasmacytoma‐bearing mice received various treatments: i.v EVs‐Dox and CD38‐EVs‐Dox (EVs‐Dox^i.v^ and CD38‐EVs‐Dox^i.v^), MN‐delivered EVs‐Dox and CD38‐EVs‐Dox (EVs‐Dox^MNs^ and CD38‐EVs‐Dox^MNs^), with PBS as the negative control (Figure S9). Treatments commenced when tumour volumes reached 20 mm^3^ and continued for 1 month. Tumour volumes were measured using a vernier caliper seven times post‐treatment initiation (Figure 6G). To assess antitumour effects, serum lactate dehydrogenase (LDH) levels, tumour volumes, Ki‐67 and proliferating cell nuclear antigen (PCNA) immunohistochemical (IHC), and P53 immunofluorescence (IF) analysis in tumour tissues were recorded. Serum LDH levels are commonly utilised to assess tumour burden in haematological malignancies, including MM.^36^ The lactic acid substrate method was used to measure the LDH levels in mice, revealing a significant decrease exclusively in the CD38‐EVs‐Dox^MNs^ group compared to the control (*p* = .0395, Figure 6H). Tumour volume increased rapidly in the PBS, EVs‐Dox^i.v^ and CD38‐EVs‐Dox^i.v^ groups. In contrast, significant inhibition was detected in the EVs‐Dox^MNs^ and CD38‐EVs‐Dox^MNs^ groups. Over 21 days, tumour growth was slower in the EVs‐Dox^MNs^ and CD38‐EVs‐Dox^MNs^ groups compared to the i.v groups. Interestingly, after 21 days, the EVs‐Dox^MNs^ group showed an increased tumour proliferation rate, whereas the CD38‐EVs‐Dox^MNs^ group exhibited a slower tumour expansion rate (Figure 6I).

At 42 days (21 days post‐final treatment, Figure 6J), the tumour volume in the CD38‐EVs‐Dox^MNs^ group was markedly reduced compared to the EVs‐Dox^i.v^ and CD38‐EVs‐Dox^i.v^ groups. This indicates the superior antitumour efficacy of CD38‐EVs‐Dox^MNs^, corroborating the in vivo distribution and targeted delivery capabilities of CD38‐EVs^MNs^ at tumour sites. IHC staining revealed that the CD38‐EVs‐Dox^MNs^ group exhibited the lowest Ki‐67 and PCNA expression compared to all other groups, indicating the most potent tumour proliferation inhibition (Figures 6K,L and S10a,c). Furthermore, Dox induces P53 protein expression, which is closely associated with cell apoptosis. Treatment with CD38‐EVs‐Dox^MNs^ significantly enhanced P53 expression in the tumour (Figure S10b,d).

The collective data demonstrate the advantages of the targeted and site‐specific antitumour efficacy of CD38‐EVs‐Dox^MNs^ in plasmacytoma‐bearing mice.

### 2.6 Biocompatibility of CD38‐EVs‐Dox^MNs^ in plasmacytoma‐bearing mice

**FIGURE 7:**
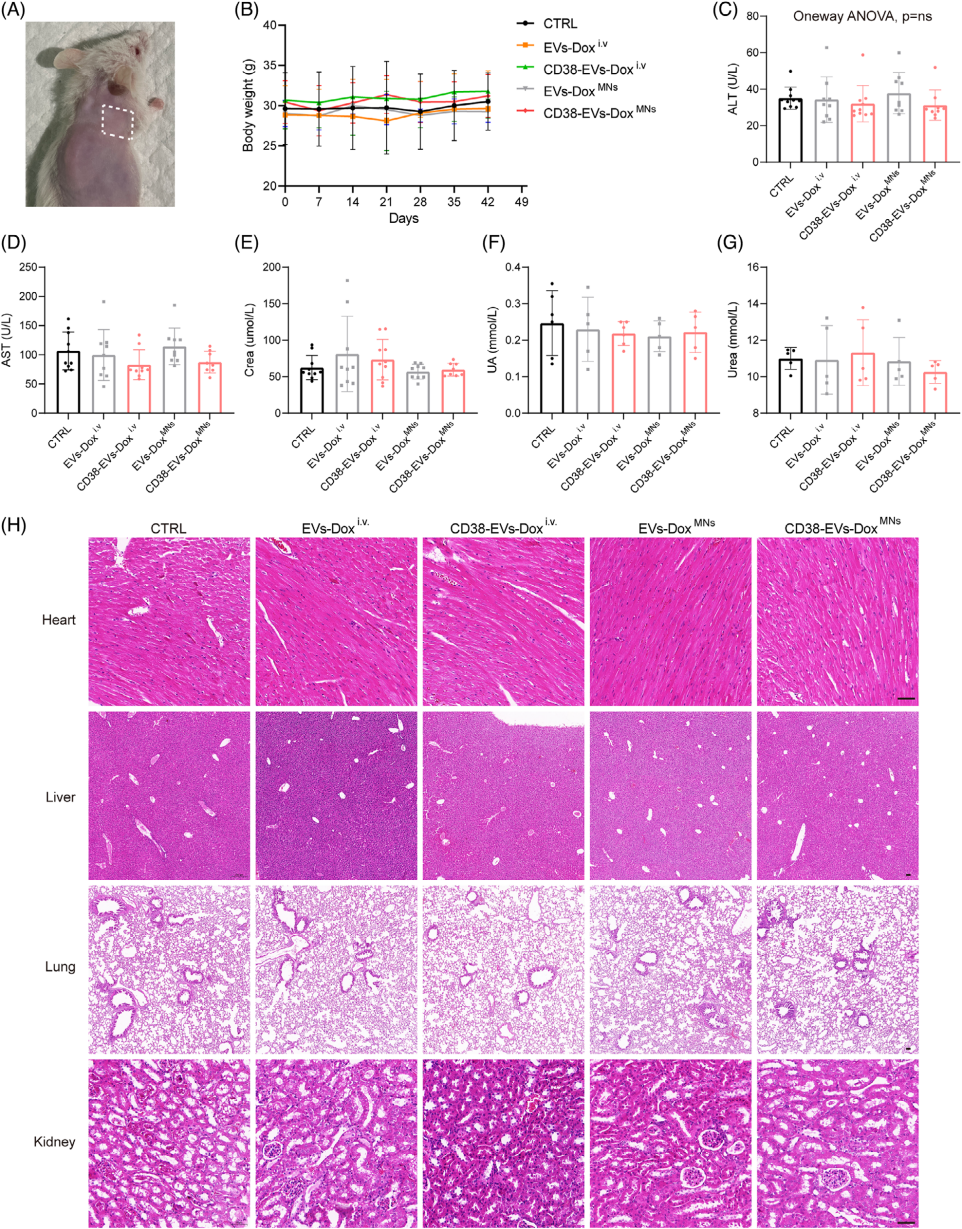
Biocompatibility of CD38‐EVs‐Dox^MNs^ in plasmacytoma‐bearing mice. (A) Photographs of naked eye view at 4 h after insertion of microneedles (MNs) into the mouse skin. (B) Mice body weight in different treatment groups. Serum levels of alanine aminotransferase (ALT) (C), aspartate aminotransferase (AST) (D), creatinine (CREA) (E), uric acid (UA) (F) and urea (G) following various treatments. (H) Representative histological images of haematoxylin and eosin (H&E) staining for heart, liver, lung and kidney tissues in different treatment groups (scale bars: 50 µm). Each bar represents the mean ± SD of 5–10 mice, *p* = ns in panels (C–G).

We evaluated the safety profile of CD38‐EVs‐Dox^MNs^ by examining skin tolerability, monitoring body weight and measuring liver and kidney function in plasmacytoma‐bearing mice. Four hours following the application of CD38‐EVs‐Dox^MNs^ to the skin of depilated mice under constant pressure, the skin pores closed naturally, showing no signs of fever, erythema, red spots, scales or blisters (Figure 7A). During the continuous 4‐h observation, the mice treated with CD38‐EVs‐Dox^MNs^ exhibited unrestricted movement, ate and drank normally, and the MNs adhered without detachment. Mice in the CD38‐EVs‐Dox^i.v^ and CD38‐EVs‐Dox^MNs^ groups consistently weighed more than those in the EVs‐Dox^i.v^ and EVs‐Dox^MNs^ groups (Figure 7B). However, no significant differences were observed across all groups at 42 days post‐treatment (Figure S11). Serum alanine aminotransferase (ALT) levels remained within normal ranges, exhibiting no significant differences across the groups (Figure 7C). The mice had elevated aspartate aminotransferase (AST) levels than the normal range, but no further increase was observed in the treated groups (Figure 7D). Serum creatinine (CREA) levels were normal in all groups except EVs‐Dox^i.v^ group, without significant differences (Figure 7E). Uric acid (UA) and urea levels showed no significant differences among the groups (Figure 7F,G).

Haematoxylin and eosin (H&E) staining was also performed on the organs (heart, liver, lungs and kidney) of mice treated with the various interventions (Figure 7H). The heart, liver and lungs showed no significant histologic changes across all groups. However, light microscopy revealed nephrotoxic alterations in kidney sections' glomeruli and uriniferous tubules in both PBS and EVs‐Dox^i.v^ groups. In the PBS group, kidney examination revealed mild glomerular mesangial proliferation, some lumen dilation and renal tubule oedema. In the EVs‐Dox^i.v^ group, renal tubular epithelial cells showed granular degeneration with protein casts in some lumens. In contrast, mice treated with CD38‐EVs‐Dox^i.v^, EVs‐Dox^MNs^ or CD38‐EVs‐Dox^MNs^ exhibited no signs of renal damage.

These findings support the favourable safety of CD38‐EVs‐Dox^MNs^ in treating plasmacytoma‐bearing mice.

## 3 DISCUSSION

Targeted therapies reduce side effects and toxicity by preventing drug accumulation at non‐target sites.^37^ Here, we successfully engineered stable CD38‐EVs that specifically target myeloma cells while minimising uptake by non‐tumour cells. Notably, CD38‐EVs^MNs^ effectively targeted tumour sites with less spleen and lung retention compared to CD38‐EVs^i.v^ and EVs^MNs^. Additionally, CD38‐EVs‐Dox^MNs^ exhibited superior antitumour efficacy and excellent biocompatibility in mice. Our findings demonstrated that MNs encapsulated with CD38‐EVs provide a more effective method for engineered EVs delivery to treat plasmacytoma in mice compared to i.v administration.

EVs are extensively utilised for targeted drug delivery,^38^ and engineering modifications, such as peptide modification, can improve their targeting capabilities.^23^, ^39^, ^40^, ^41^ FDA‐approved PEG has been shown to extend the blood circulation time of EVs.^42^ We showed that CD38‐EVs effectively targeted myeloma cells both in vitro and in vivo. Interestingly, in co‐culture models, there was no significant difference in the uptake of standard EVs and CD38‐EVs by myeloma cells. In contrast, non‐tumour stromal cells demonstrated a significantly greater uptake of both types of EVs when compared to the tumour cells. The myeloma microenvironment consists of tumour cells and various stromal cells such as BMSCs, fibroblasts and vascular endothelial cells.^43^ EV uptake rates vary by cell type and condition due to different internalisation mechanisms.^44^ The markedly high uptake rates of our two types of EVs in non‐tumour cells might explain the lack of significant differences in uptake efficiency between EVs and CD38‐EVs in myeloma cells in the co‐culture systems. Importantly, CD38‐EVs exhibited substantially reduced uptake by non‐tumour stromal cells than standard EVs, demonstrating their potential safety for delivering chemotherapeutic agents by targeting tumour cells and sparing non‐tumour cells. This is corroborated by our in vivo data.

The distribution of EVs throughout the body is influenced by their method of administration.^13^, ^45^ Following i.v injection, EVs primarily localise in the liver and spleen, with limited accumulation in tumour tissues, especially those distant from highly vascularised regions.^12^ We have previously observed that EVs derived from MSCs (MSC‐EVs) predominantly accumulate in the spleen, liver and lungs of C57BL/6J mice following i.v or intraperitoneal administration.^4^, ^30^ We designed CD38‐EVs for targeting tumour cells in mice via i.v injection and assessed their biodistribution in normal and tumour mouse models. However, neither CD38‐EVs nor EVs effectively accumulated at the tumour site. Alternatively, the accumulation of EVs and CD38‐EVs was predominantly observed in the lungs and spleen, aligning with the results of our previous studies^4^, ^30^, ^31^ and Wiklander et al.'s,^45^ in which the majority of administrated EVs are captured by the mononuclear phagocyte system (MPS). Despite their benefits over synthetic delivery systems, such as lower immunogenicity, biocompatibility and better barrier permeability, EV's distribution beyond the liver and spleen is limited by MPS uptake.^46^ Consequently, researchers are developing engineered EVs incorporating targeting peptides such as the cardiac homing peptide to improve delivery to cardiac myocytes.^47^ However, these EVs also primarily accumulate in the liver and spleen.^48^ Liu et al.^49^ demonstrated that pre‐injecting blocking EVs can inhibit the uptake of EVs by the liver and spleen, enhancing myocardial protection from Dox with subsequent therapeutic EV injections. Our study suggests that minimising non‐specific CD38‐EVs accumulation in the lungs and spleen is critical for better‐targeting delivery of them to tumour sites. Additionally, since we reported that intravenously administered EVs could increase the risk of fatal pulmonary embolism in mice,^31^ exploring alternative delivery methods for CD38‐EVs beyond i.v routes is essential.

MNs offer a novel and effective method for the transdermal administration of EVs.^50^ In situations where EVs need to function at specific sites, particularly within superficial body tissues, using EV‐loaded MNs represents an optimal solution. Empirical studies have demonstrated the efficacy of MNs in delivering EVs for the treatment of diverse diseases, as well as in enhancing tissue repair and regeneration.^51^, ^52^, ^53^, ^54^, ^55^, ^56^, ^57^ However, the biodistribution, targeting efficiency, biological safety, and treatment efficacy of engineered EVs delivered via MNs compared to i.v administration remain unknown. In our plasmacytoma‐bearing NOD/SCID mouse models, we employed MNs to deliver CD38‐EVs, which exhibited improved targeting and delivery to tumour sites compared to standard EVs. It has been indicated that positively charged nanocarriers may possess superior skin penetration capabilities.^58^ The engineered CD38‐EVs acquired a positive surface charge, which likely enhanced their access to plasmacytoma sites. Both MNs and CD38‐EVs‐Dox^MNs^ are biocompatible with skin and internal organs, with minimal EV distribution in the lungs compared to i.v administration. This is likely due to the ability of CD38‐EVs^MNs^ to bypass pulmonary capillary filtration. Furthermore, the PEGylation strategy employed in our study may have modulated biodistribution by reducing non‐specific interactions with endothelial cells in the lung microvasculature, thereby minimising pulmonary retention.^59^ Given that the high doses of intravenously administered EVs increase the risk of fatal pulmonary embolism in mice,^31^ CD38‐EVs‐Dox^MNs^ offer a safer alternative of delivery system for targeted EV delivery. CD38‐EVs‐Dox^MNs^ showed superior inhibition of tumour growth and favourable biocompatibility compared to non‐targeted EVs‐Dox^MNs^ and CD38‐EVs‐Dox^i.v^. These effects should be attributed to CD38‐EVs' selective targeting and MNs' ability to avoid EVs distribution in MPS (lungs and spleen). The findings demonstrate that MNs are superior to i.v administration for delivering CD38‐EVs‐Dox in plasmacytoma therapy. As MNs facilitate CD38‐EVs‐Dox to target tumour sites in tumour mice locally and spread through lymphatic channels and capillaries, further investigations are needed to understand the impact of our CD38‐EVs‐Dox^MNs^ on tumour sites in various tissues like the parabrachial bone, lymph nodes and spine. Additionally, considering the immunodeficient background of NOD/SCID mice, potential variations in monocyte/macrophage abundance and function could affect EV distribution. Future research utilising immunocompetent cancer models or humanised mouse models could offer a deeper insight into the immune factors that govern EV distribution and their therapeutic effectiveness. Moreover, while umbilical cord MSC‐derived EVs are widely utilised in preclinical studies due to their low immunogenicity and therapeutic properties, species‐specific differences in receptor‒ligand interactions may impact EV uptake and targeting in murine cells. Further research with syngeneic EVs or humanised mouse models would be valuable to determine whether the targeting efficiency observed in this study holds in a more physiologically relevant immune environment.

MNs are increasingly being explored for systemic therapies beyond localised applications. Clinical trials on MN‐base delivery of influenza and SARS‐CoV‐2 vaccines have shown efficacy similar to intramuscular injections.^60^, ^61^ In addition to vaccines, phase I trials of adalimumab‐loaded MNs for rheumatoid arthritis (NCT03607903) have demonstrated their feasibility for treating chronic systemic diseases. While MN‐based transdermal delivery for systemic cancer therapy is still in its nascent stages, further studies are required to refine dosing strategies and evaluate the long‐term therapeutic efficacy. Subcutaneous anticancer agents such as bortezomib and rituximab, traditionally administered via injection, could be adapted for MN delivery to reduce pain and improve patient compliance. Building on these concepts, our CD38‐EVs^MNs^ targeting system holds the potential to encapsulate a range of therapeutic agents, including proteasome inhibitors and cytotoxic drugs, for the treatment of systemic MM. Moreover, previous studies have investigated the synergistic effects of CAR‐T cells and bispecific antibodies with chemotherapeutic agents, such as Dox and liposomal mitoxantrone.^62^ Leveraging these findings, our engineered CD38‐EVs‐Dox^MNs^ may enhance MM treatment efficacy when combined with standard regimens, including proteasome inhibitors or immunomodulatory drugs. In clinical applications, factors such as tumour location, depth and skin permeability may influence the precision and effectiveness of MN‐mediated delivery. Therefore, further studies utilising models that more closely mimic human tumour environments, such as patient‐derived xenografts or larger animal models with more complex skin structures, are essential to more comprehensively assess the effectiveness and translational potential of MN‐based EV delivery.

In summary, this study showed that CD38‐EVs, targeting myeloma cells and reducing the uptake by non‐tumour cells and tissues, along with CD38‐EVs‐Dox^MNs^, offer a non‐invasive, site‐specific and effective treatment for plasmacytoma with favourable safety and histocompatibility compared to intravenously delivered CD38‐EVs‐Dox. As an emerging delivery system, the tissue distribution, safety and therapeutic potential of MNs loaded with engineered EVs deserve further investigation in preclinical studies for other diseases and clinical studies. Additionally, while our EV isolation protocol ensures batch consistency at the laboratory scale, future studies should explore strategies to overcome the challenges of industrial‐scale production. Moreover, the clinical translation of MNs and their industrial‐scale manufacturing necessitates a cost‒benefit analysis in comparison to conventional systemic administration routes. Scalable production technologies, such as 3D printing and micro‐moulding, are improving cost‐effectiveness and minimising material waste.^63^, ^64^, ^65^, ^66^ However, future investigations are required to assess whether the therapeutic advantages of MNs outweigh the associated fabrication costs.

## 4 MATERIALS AND METHODS

### 4.1 Ethical regulations

The research conducted in this study adheres to all ethical regulations. Human umbilical cord specimens were procured with informed consent from patients undergoing cesarean delivery at full‐term gestation (38‒40 weeks). The study received approval from the Independent Ethics Committee of Union Hospital, Tongji Medical College, Huazhong University of Science and Technology ([2023] (0584‐01)), and adhered to the ethical principles outlined in the Declaration of Helsinki. The animal experiments were performed under guidance of the Ethical Conduct in the Care and Use of Animals. Our study received approval from the Institutional Animal Care and Use Committee of Tongji Medical College, Huazhong University of Science and Technology (number: 3770). The tumour size did not exceed the maximum allowable volume of 1500 mm^3^, as stipulated by the ISCIII Ethical Committee, when applicable.

### 4.2 Cell culture

MSCs derived from umbilical cord samples were isolated and characterised as previously described.^67^ To avoid potential interference from EVs derived from foetal bovine serum (FBS; Gibco), the FBS was subjected to high‐speed centrifugation at 16 000 *g* for 60 min at 4°C using an Avanti J‐26S XPI High‐Performance Centrifuge (Beckman Coulter) to effectively remove EVs prior to its use in the preparation of complete culture medium for umbilical cord MSCs. MSCs were cultured in complete Dulbecco's modified Eagle's medium (DMEM/F12; Gibco) under standard conditions of 37°C and 5% CO_2_.

The RPMI8226, U266 and HUVECs cell lines were purchased from Pricella, Inc. Human BMSCs were purchased from Cyagen Biosciences Inc. The human chronic myeloid leukaemia K562 cell line was routinely preserved in our laboratory. All cell lines were verified using STR profiling and confirmed to be free from mycoplasma contamination. The RPMI8226, U266 cells and K562 were cultured in complete RPMI1640 (Gibco) medium. The HUVECs cell line was grown in ECM medium containing an endothelial cell growth medium bullet kit (Sciencell). BMSCs were cultured in complete α‐MEM (Hyclone) medium.

### 4.3 Synthesis of DSPE‐PEG (2000)‐CD38 peptide conjugates

CD38 peptide (ARGDYYGSNSLDYW), FITC‐labelled CD38 peptide and phospholipid‒polyethylene glycol‒N‐hydroxysuccinimide (DSPE‐PEG‐NHS, PEG molecular weight 2000) were synthesised by Xi'an ruixi Biological Technology Co. A total of 100 mg of DSPE‐PEG‐NHS (PEG molecular weight 2000) was dissolved in 3 mL of dimethyl formamide (DMF). To this solution, CD38 peptide (1.1 equivalents) and triethylamine (3.0 equivalents) were subsequently added, ensuring complete dissolution. The reaction mixture was transferred to a dialysis bag with a 2000 Da molecular weight cutoff and dialysed in ultrapure water for 24 h. The dialysate was collected and the product obtained by freeze‒drying was phospholipid‒polyethylene glycol‒CD38 conjugates (DSPE‐PEG‐CD38). 1H‐NMR spectrum of the purified product was obtained in a Bruker AVANCE III NMR spectrometer (Bruker Biospin AG, Fallanden).

### 4.4 Receptor expression profiling and binding assays

RPMI8226 and U266 cell lines were subjected to membrane staining utilising a PKH26 Red Fluorescent Cell Linker Kit (Sigma). Subsequently, these stained cells were plated into 12‐well plates and co‐incubated with FITC‐conjugated CD38 antibody (BD Biosciences) and FITC‐labelled CD38 peptide for 30 min. Following dual PBS (Gibco) washes, cellular analysis was conducted using an inverted fluorescence microscope (Olympus). Cell lines K562 and MSCs were employed as negative controls within the experimental framework.

### 4.5 EVs isolation

According to our previous publications, MSCs supernatants were employed for EVs isolation.^4^, ^30^ Umbilical cord‐derived MSCs were incubated in EV‐depleted medium for 48 h, after which the supernatant was harvested for EV isolation. The isolation process entailed a sequential centrifugation protocol applied to the culture medium. Initially, the culture medium was first subjected to centrifugation at 750 *g* for 15 min, and followed by a second centrifugation step at 2000 *g* for 20 min. The clarified supernatant was then subjected to high‐speed centrifugation at 16 000 *g* for 60 min using an Avanti J‐26S XPI High‐Performance Centrifuge (Beckman Coulter) to pellet the EVs. Post‐centrifugation, the EVs were resuspended in PBS, then either utilised promptly or preserved at −80°C for future use. The yield of MSC‐derived EVs was ascertained by lysing the purified vesicles in RIPA buffer (Beyotime) and measuring the total protein concentration using a bicinchoninic acid (BCA) protein assay (Beyotime).

### 4.6 EV labelling assays

For in vitro EV internalisation studies, purified EVs were labelled with the PKH26 Red Fluorescent Cell Linker Kit (Sigma). The EV pellet was resuspended in 500 µL of Diluent C, followed by the addition of 2 µL of PKH26 ethanolic dye solution, mixed in an equal volume of Diluent C and incubated at 25°C for 5 min. Unbound PKH26 dye was subsequently removed using an Amicon ultracentrifugal filter with a 100 kDa membrane (Millipore).

For carboxyfluorescein diacetate succinimidyl ester (CFSE; Invitrogen) labelling, 500 µg of EVs in 1 mL PBS were combined with 1 µM CFSE and incubated at 25°C in the dark for 10 min. The reaction was terminated by adding precooled DMEM/F12 complete medium, followed by unbound dye removal via an Amicon Ultra 100 kDa filter.

For in vivo biodistribution analysis, EVs were labelled with the DIR dye (Rengen Biosciences). A mixture of 5 µL DIR (220 µg/mL) and 500 µg EVs in 100 µL PBS was incubated at 25°C for 30 min. Unbound DIR was removed using an Amicon Ultra 100 kDa filter.

### 4.7 Engineered EVs preparation

CD38‐EVs were prepared as follows. A quantity of 250 µg of EVs (BCA protein quantification) was added with 25 µL of DSPE‐PEG‐CD38 solution (1 µg/µL), and the mixture was gently pipetted to ensure homogeneity. The mixture was allowed to incubated at 25°C for 1 h, after which it was transferred to centrifuge tubes and centrifugated at 16 000 *g* for 1 h. The supernatant was aspirated to remove unbound DSPE‐PEG‐CD38, and the resulting pellet was identified as CD38‐EVs. The FITC‐labelled anti‐CD38pep antibody, synthesised by Baiqi Biotechnology Co., Ltd., was observed binding with CD38‐EVs using a fluorescence microscope.

The preparation of CD38‐EVs‐Dox was executed as delineated hereunder. An amount of 1 mg of Dox (Abmole) was solubilised in 1.7242 mL of dimethyl sulphoxide (Amresco), yielding a storage solution with a molarity of 1 mM. A measure of 30 µg of Dox was then combined with EVs derived from 4 × 10^7^ MSCs and the mixture was co‐incubated at 37°C for 1 h. Following incubation, non‐internalised Dox was separated from the EVs by centrifugation at 16 000 *g* for 1 h. The sediment obtained post‐centrifugation was designated as EVs‐Dox, which was subsequently dissolved in PBS for further utilisation. The synthesis of CD38‐EVs‐Dox adhered to the identical protocol established for CD38‐EVs, with the sole distinction being the substitution of EVs with EVs‐Dox in the preparation process.

### 4.8 Liquid chromatography–tandem mass spectrometry

The CD38‐EVs‐Dox was processed by the RIPA lysis buffer for 15 min, after which methanol was added in a 9:1 ratio. Subsequently, the mixture was then vortexed for 2 min and then centrifuged at 12 000 *g* for 10 min at 4°C. The supernatant was then evaporated and redissolved with 100 µL of 50% methanol, after which LC–MS/MS was performed. Liquid chromatography was performed using an ACQUITY Premier Ultra‐Performance Liquid Chromatography (UPLC) System (Waters). Chromatography was performed on an ACQUITY UPLC HSS T3 column (2.1 × 100 mm, 1.7 µm) (Waters) with a mobile phase A consisting of 75% acetonitrile (containing .05% trifluoroacetic acid) and a mobile phase B consisting 25% water (containing .05% trifluoroacetic acid). The flow rate was set at .20 mL/min, with the column temperature maintained at 35°C and the detection wavelength configured to 260 nm.

### 4.9 Fabrication of MNs encapsulated with engineered EVs

EVs‐Dox and CD38‐EVs‐Dox were dispersed separately in deionised water to prepare the vesicular aqueous solution with a concentration of 1 g/L. One gram of gelatin was dissolved in 9 mL of deionised water and stirred at 60°C to achieve complete dissolution and uniform homogenisation. At 40°C, the gelatin solution was combined with the vesicular solution in a 4:1 volume ratio to yield a homogeneous precursor solution. The specifications for the polydimethylsiloxane MN mould were as follows: a needle length of 600 µm, a base diameter of 280 µm, a needle tip spacing of 600 µm, a base groove depth of 2.5 mm and an array configuration of 15 × 15, resulting in overall dimensions of 11 × 11 mm^2^. Then, 200 µL of the precursor solution was deposited into the MNs moulds, vacuumed to ‒.06 MPa, maintained in a vacuum environment for 30 min to eliminate any bubbles, and the moulds were placed in a 40°C atmosphere for 1 h to remove the water by drying and to obtain solid‐form MNs encapsulated with EVs‐Dox and CD38‐EVs‐Dox. Next, the second gelation solution without containing EVs‐Dox or CD38‐EVs‐Dox was applied to the MN mould to form the base, which was then dried in a clean oven at 40°C. Finally, the MNs loaded with either EVs‐Dox or CD38‐EVs‐Dox were carefully removed from the mould, sealed in an aluminium foil bag and preserved at 4°C for further use.

To investigate the biodistribution of EVs and CD38‐EVs delivered by MNs in vivo, we engineered MNs encapsulating EVs and CD38‐EVs labelled with DiR fluorescent dye (Rengen Biosciences) and PKH26. The methodology employed was analogous to that described above.

### 4.10 NTA

The morphological parameters and particulate concentration of the EVs, CD38‐EVs and CD38‐EVs‐Dox were quantitatively analysed using NTA on the ZetaView platform (Particle Metrix). The aforementioned EVs were resuspended in PBS and analysed at room temperature following appropriate dilution. The detection process involved an amplification of the scatter plot by a decuple factor to facilitate the visual capture of these EVs, thereby enabling the observation of their Brownian motion.

### 4.11 TEM

EVs, CD38‐EVs and CD38‐EVs‐Dox (10 µL in volume) were applied separately to a 200‐mesh copper grid and left to adhere for 2 min. The samples were dehydrated using absorbent filter paper and uniformly coated with 10 µL of 2% uranyl acetate solution to enhance contrast. After being dried and kept in the dark for 3 min, the grids were visualised using an HT7800 TEM (Hitachi).

### 4.12 SEM

MN specimens were meticulously affixed onto conductive adhesive tape and then subjected to a sputtering process using a Quorum SC7620 Sputter Coater (Quorum). Gold deposition was performed for 45 s under a constant current of 10 mA. Subsequently, the SEM observations were employed by a Tescan MIRA LMS (Tescan) to capture the morphological features of the samples and perform energy‐dispersive X‐ray spectroscopy (EDS) mapping. The morphology imaging was executed at an acceleration voltage of 3 kV, whereas the EDS mapping was performed at 15 kV using the SE2 secondary electron detector.

### 4.13 LSCM

EVs and CD38‐EVs labelled with CFSE (Solarbio) were incubated with RPMI8226 and U266 cells in culture media for 8 h. Following incubation, the cells were first fixed with 4% paraformaldehyde at room temperature for 15 min, then permeabilised with .5% Triton X‐100 for 5 min. The cytoskeleton was subsequently stained with rhodamine‐phalloidin (Yeasen) for 30 min, and the nuclei were counterstained with 4',6‐diamidino‐2‐phenylindole (DAPI) (Solarbio) for 10 min. Finally, the uptake and intracellular localisation of EVs and CD38‐EVs in myeloma cells were observed under a Zeiss LSM 900 Airyscan laser scanning confocal microscope (Carl Zeiss).

PKH26 fluorescent dye was integrated homogeneously with a gelatin solution to fabricate PKH‐26‐labelled MNs. The fluorescent images of MNs were acquired using a TCS SP8 LSCM (Leica).

### 4.14 Flow cytometry

The analysis of the surface marker CD38pep of EVs and CD38‐EVs was carried out using Nano Flow Cytometry (Nano FCM). Fluorescent submicrometer beads (.2, .5 and .76 µm) (Bangs Laboratories, Fishers) were used to define the EVs gate. In this study, purified EVs were characterised as events gated with diameters less than .76 µm. To assess the proportion of CD38‐EVs, the EVs and CD38‐EVs samples were incubated with FITC‐labelled anti‐CD38pep antibody. The resulting data were then processed and analysed using FlowJo software (Tree Star).

CD38 expression in RPMI8226, U266, HUVEC and BMSCs was analysed by FCM. A total of 1 × 10^5^ cells were incubated with 5 µL FITC‐conjugated anti‐CD38 antibody (BD Biosciences) for 20 min. The cells were then washed and resuspended in 150 µL PBS. The Sony ID7000 instrument (Sony), NovoCyte D3000 FCM instrument (Aisen) and FlowJo software were used in this study.

In order to assess the internalisation kinetics of CD38‐EVs by myeloma cells in vitro, PKH26‐labelled EVs and CD38‐EVs were separately co‐cultures with 1 × 10^5^ RPMI8226 cells and 1 × 10^5^ U266 cells within culture media. This incubation occurred with time intervals of 4, 8 and 12 h. The uptake rate was quantitatively assessed utilising the Sony ID7000 FCM instrument (Sony) and FlowJo software.

To determine the efficiency of PKH26‐labelled CD38‐EVs uptake by myeloma cells and tumour‐infiltrating stromal cells, co‐culture models of myeloma cells with BMSCs and HUVECs were constructed separately. RPMI8226 cells and U266 cells stained with CFSE were individually plated in each well of a 12‐well plate, which had been pre‐seeded with 2 × 10^4^ BMSCs/well and 2 × 10^4^ HUVECs/well 24 h before the addition of myeloma cells. The co‐culture cells were incubated with PKH26‐labelled EVs and PKH26‐labelled CD38‐EVs for 12 h. The adherent BMSCs and HUVECs were detached from the plates using .25% trypsin‐EDTA (Invitrogen) and collected with RPMI8226 and U266. Finally, both myeloma and stromal cells were analysed using an FCM instrument and the data were analysed with FlowJo software.

To further evaluate the binding capacity of CD38‐EVs binding to CD38 antigen, 1 × 10^5^ U266 cells were first treated with CD38 peptide (Ruixi) for 4 h to block surface CD38, followed by an 8‐h incubation with PKH26‐labelled CD38‐EVs. The uptake rate was quantitatively analysed using the NovoCyte D3000 flow cytometer (Aisen) and Novoexpress software.

### 4.15 Western blot

MSCs and EVs were incubated in RIPA for 30 min to facilitate lysis. The resulting lysates, containing 25 µg of EV protein and 30 µg of MSC protein, were subjected to 10% sodium dodecyl sulphate−polyacrylamide gel electrophoresis (Sigma) and subsequently transferred onto nitrocellulose membranes (Merck Millipore) for further analysis. The membranes were subsequently incubated with primary antibodies targeting Annexin A1 (Cell Signaling Technology; 1:1000), Alix (Cell Signaling Technology; 1:1000), TSG101 (Abcam; 1:1000), CD9 (Abcam; 1:1000), Calnexin (Proteintech; 1:5000) and GAPDH (Cell Signaling Technology; 1:3000). Finally, the membranes were treated with horseradish peroxidase (HRP)‐conjugated secondary antibodies (Cell Signaling Technology; 1:2000) for detection.

### 4.16 In vitro cytotoxicity assays

Cell cytotoxicity was assessed using the CCK‐8 kit (Dojindo) following the protocol. Myeloma cells were cultured in triplicate at an appropriate density in 96‐well plates 6 h before each experiment. A total of 10 µg/mL of EVs, CD38‐EVs, EVs‐Dox, and CD38‐EVs‐Dox were added separately to myeloma cells for 24, 48 or 72 h. Absorbance was quantified at 450 nm using a microplate reader (PerkinElmer EnSpire).

### 4.17 EdU incorporation assay

To assess cell proliferation, the Cell‐Light EdU Apollo 567 In Vitro Imaging Kit (Ribobio) was employed. Briefly, the cells were plated in 24‐well plates at a density of 1 × 10^5^ cells per well and incubated overnight. After incubation with 10 µg/mL EVs, EVs‐Dox and CD38‐EVs‐Dox for 24 h, the cells were labelled with 10 µM EdU for 2 h. The cells were fixed in 4% paraformaldehyde for 30 min, stained with a fluorescent dye, and then washed twice with PBS and resuspended in .5% Triton X‐100 in PBS. Nuclei were counterstained with Hoechest33342 (Beyotime). EdU‐positive cells were visualised and quantified using an inverted fluorescence microscope.

### 4.18 Mice

Male C57BL/6J (6−8 weeks old) and male NOD/SCID (6−8 weeks old) mice were obtained from Huafukang Biotechnology Co., Ltd. The animals were housed and bred under specific pathogen‐free conditions, with a 12‐h light/12‐h dark cycles, and were provided with ad libitum access to a standard chow diet and water in the animal care facility.

A subcutaneous plasmacytoma model was established by injecting RPMI8226 cells (5 × 10^6^ cells per mouse) subcutaneously into sixty 6‐week‐old NOD/SCID mice. Tumour volume was measured using calipers (volume = .5 × length × [width]^2^).

The determination of animal sample sizes in this study was guided by the resource equation approach and ethical considerations to ensure statistical robustness while minimising unnecessary animal use. According to the resource equation approach, an appropriate sample size for in vivo biodistribution studies typically ranges from four to six animals per group. For the in vivo antitumour efficacy experiments, five experimental groups were established, with the recommended sample size per group ranging from three to five animals, as calculated based on the same approach. All experimental procedures were carefully planned and carried out in full compliance with the 3R principles (replacement, reduction, and refinement).

### 4.19 Biodistribution of EVs in vivo

For biodistribution studies of intravenously administrated EVs and CD38‐EVs in normal mice, six 8‐week‐old C57BL/6J mice were distributed randomly into two groups and treated intravenously via tail vein injection with 50 µg of PKH26 labelled EVs and CD38‐EVs. Mice were killed 1 and 3 h after EVs and CD38‐EVs injection, and ex vivo organs were harvested. Slides were observed and captured via an inverted fluorescence microscope.

We assessed the sensitivity of FMI in detecting EVs. After injecting EVs via the tail vein at a protein concentration of 50 µg, we conducted imaging using the Lago/Lago X imaging system (Spectral Instruments Imaging).

To investigate the biodistribution of EVs and CD38‐EVs in model mice, the two types of EVs were administered 50 µg of DiR‐stained EVs and CD38‐EVs intravenously and via MNs, respectively, to plasmacytoma‐bearing NOD/SCID mice. One and 3 h after treatment, the mice were imaged using an FMP Image System (Britton Chance Center for Biomedical Photonics). The scanning procedure was conducted in accordance with the methodology described in our previously published abstract.^32^ In addition, 50 µg of EVs and CD38‐EVs labelled with PKH26 were applied in the same manner as described above. Three hours after application, the extracted organs (spleen and lung) and tumour tissues were harvested and observed under an inverted fluorescence microscope.

### 4.20 In vivo antitumour effects and toxicity assessments

To further investigate the antitumour effects and toxicity profiles of various treatments in plasmacytoma‐bearing NOD/SCID mice, the model animals were randomly assigned to one of five treatment groups (*n* = 10 per group) when the tumour volume reached 20 mm^3^. The treatments administered were: (1) control (PBS), (2) EVs‐Dox by i.v injection (EVs‐Dox^i.v^), (3) CD38‐EVs‐Dox by i.v injection (CD38‐EVs‐Dox^i.v^), (4) EVs‐Dox loaded by MNs (EVs‐Dox^MNs^), and (5) CD38‐EVs‐Dox loaded by MNs (CD38‐EVs‐Dox^MNs^). The mice in the EVs‐Dox^i.v^ group and the CD38‐EVs‐Dox^i.v^ group were injected with 50 µg per mouse twice a week for 4 weeks. The mice in the EVs‐Dox^MNs^ group and the CD38‐EVs‐Dox^MNs^ group were applied with MNs containing 50 µg of EVs‐Dox and CD38‐EVs‐Dox twice a week for 4 weeks. Tumour progression in the five treatment groups was monitored weekly for 7 weeks using vernier calipers, while the body weights of the mice were also recorded at each time point. On the 28th day, approximately 200–500 µL of blood was collected and assessed for hepatic and renal function (ALT, AST, CREA, UA and urea) and LDH. The mice were sacrificed and specimens of the tumours were fixed and pathologically evaluated for treatment efficacy. On the 42nd day, the mice were euthanised, and tissue samples from the heart, liver, lung and kidney were collected and fixed for subsequent pathological analysis.

### 4.21 Histological, IHC and IF analysis

H&E staining was carried out as previously described.^4^ For IHC staining, tumour tissue sections were incubated with Ki‐67 antibodies (Abcam; 1:1000) and PCNA antibodies (Proteintech; 1:1000), followed by a blocking step using bovine serum albumin (Sigma). Following incubation with primary antibodies, tissue sections were treated with HRP‐conjugated secondary antibodies (Abcam; 1:2000) for 1 h, followed by HRP‐DAB (Abcam; 1:5000) chromogenic staining. The slides were then counterstained with haematoxylin, dehydrated and mounted. For IF staining, anti‐P53 antibody (Abclonal; 1:100) was employed as the primary antibody, and goat anti‐rabbit IgG‐FITC (Abcam; 1:500) served as the secondary antibody. Nuclei were counterstained with Hoechst 33258 (Beyotime). Fluorescent signals were visualised and captured using a fluorescence microscope.

### 4.22 Statistical analysis

All statistical analyses were conducted using GraphPad Prism version 9.0 (GraphPad Software) and results are presented as the mean ± standard deviation (SD). For comparisons between two groups, Student's *t*‐test was applied, while one‐ or two‐way analysis of variance (ANOVA) was used for comparisons involving more than two groups. If the data followed a normal distribution, ANOVA was employed. In cases of non‐normal distribution, the Kruskal‒Wallis one‐way ANOVA test was utilised, followed by the Tukey's post hoc test for multiple comparisons. Sample sizes are specified in the figure legends. *p*‐Value <.05 was considered statistically significant; ^*^ *p* < .05; ^**^ *p* < .01; ^****^ *p* < .0001; ns, non‐significant.

## AUTHOR CONTRIBUTIONS

*Writing—original draft, methodology, investigation, formal analysis, data curation and conceptualisation*: Yulin Cao. *Validation, methodology, investigation, formal analysis, data curation and conceptualisation*: Xuan Hu. *Methodology, investigation, formal analysis and data curation*: Di Wu. *Methodology, formal analysis and data curation*: Yuxuan Jiang. *Validation, methodology and formal analysis*: Yali Yu and Shan Wang. *Methodology, investigation and data curation*: Wenlan Chen. *Investigation and formal analysis*: Yaoying Long and Jiao Qu. *Validation*: Liuyue Xu. *Methodology and data curation*: Bianlei Yang. *Writing—review and editing*: Blal Chakhabi. *Resources and validation*: Hongxiang Wang. *Methodology, resources and validation*: Yong Deng. *Writing—review and editing, supervision and conceptualisation*: Lei Chen. *Supervision, resources, project administration and funding acquisition*: Zhichao Chen. *Writing—review and editing, writing—original draft, supervision, project administration, funding acquisition and conceptualisation*: Qiubai Li.

## CONFLICT OF INTEREST STATEMENT

The authors declare they have no conflicts of interest.

## ETHICS STATEMENT

This study and included experimental procedures were approved by the Independent Ethics Committee of Union Hospital, Tongji Medical College, Huazhong University of Science and Technology ([2023] (0584‐01)), and followed the principles in the Declaration of Helsinki. The animal experiments were performed according to the guidelines for Ethical Conduct in the Care and Use of Animals, as stated in The International Guiding Principles for Biomedical Research involving Animals, developed by the Council for International Organizations of Medical Sciences (CIOMS) and were approved by the Institutional Animal Care and Use Committee of Tongji Medical College, Huazhong University of Science and Technology (IACUC number: 3770).

## Supporting information



Supporting Information

Supporting Information

Supporting Information

Supporting Information

Supporting Information

Supporting Information

Supporting Information

Supporting Information

Supporting Information

Supporting Information

Supporting Information

Supporting Information

Supporting Information

## Data Availability

Data sharing not applicable to this article as no datasets were generated or analysed during the current study.
