# Supplementary material for: Targeting capacity, safety and efficacy of engineered extracellular vesicles delivered by transdermal microneedles to treat plasmacytoma in mice
Source: Clin Transl Med. 2025 May 2;15(5):e70327. doi: 10.1002/ctm2.70327 (PMC12048306; doi:10.1002/ctm2.70327)
Supplement: Supplementary file 1 — Supporting Information [file CTM2-15-e70327-s005.docx]

**Supplementary Information**

**Targeting capacity, safety and efficacy of engineered extracellular vesicles delivered by transdermal microneedles to treat plasmacytoma in mice**

**Contents**

| Fig. S1 | Identification of antagonist peptide for CD38 |
| --- | --- |
| Fig. S2 | Synthesis and characterization of DSPE-PEG-CD38 |
| Fig. S3 | Uptake of EVs targeted with varying densities of CD38pep |
| Fig. S4 | CD38 expression in various cell lines |
| Fig. S5 | Targeting capability of CD38-EVs following CD38 antigen blockade |
| Fig. S6 | FMI fails to detect tiny amounts of EVs in mice and successful construction of the FMP imaging system |
| Fig. S7 | Biodistribution of EVs and CD38-EVs in the heart, liver, kidney and brain |
| Fig. S8 | Construction and characterization of CD38-EVs-Dox |
| Fig. S9 | Schematic illustration of MNs loaded with CD38-EVs-Dox |
| Fig. S10 | Antitumor effect of different treatment groups *in vivo*. |
| Fig. S11 | Statistical analysis of body weights in all treatment groups at 42 days |
| Table. S1 | Quantification of EVs extracted from umbilical cord MSCs culture medium by high-speed centrifugation |
| Table. S2 | Determination of doxorubicin loading capacity of CD38-EVs |


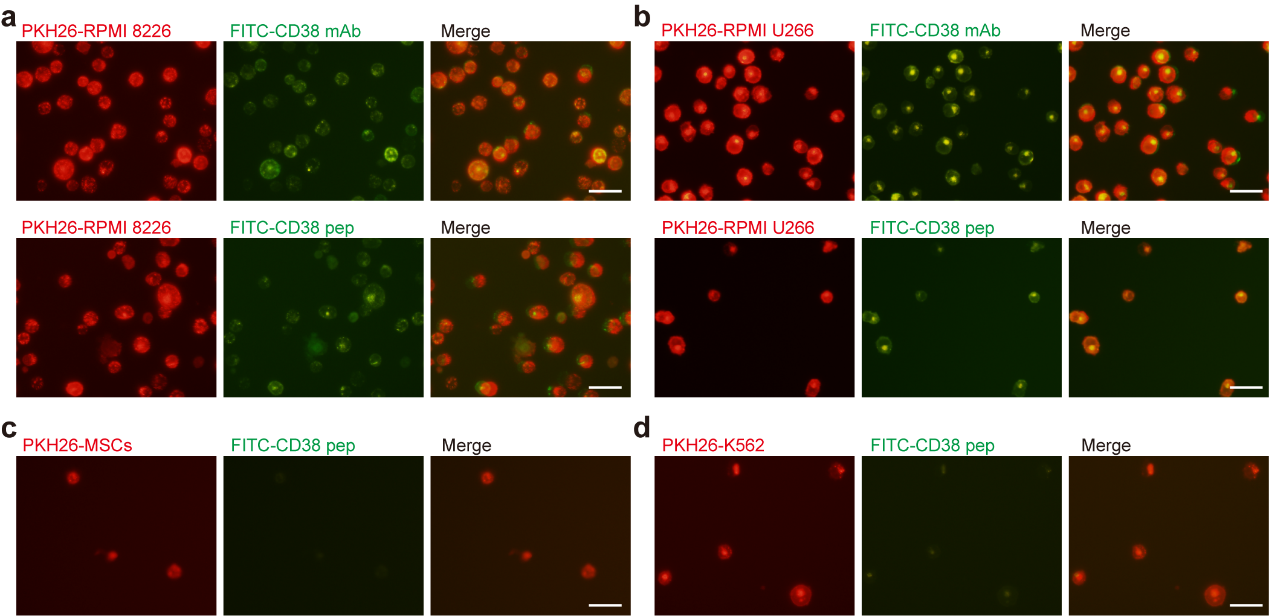


**Fig. S1. Identification of antagonist peptide for CD38.** Cellular binding assays using FITC-labeled CD38pep in CD38^high^ myeloma cell lines of RPMI8226 (a) and U266 (b), and CD38-negative MSCs (c) and K562 cell. (d). FITC-labeled CD38 monoclonal antibody (mAb) used as a positive control. Binding was detected by an inverted fluorescence microscope (*n* = 3) (Scale bars, 100 μm).


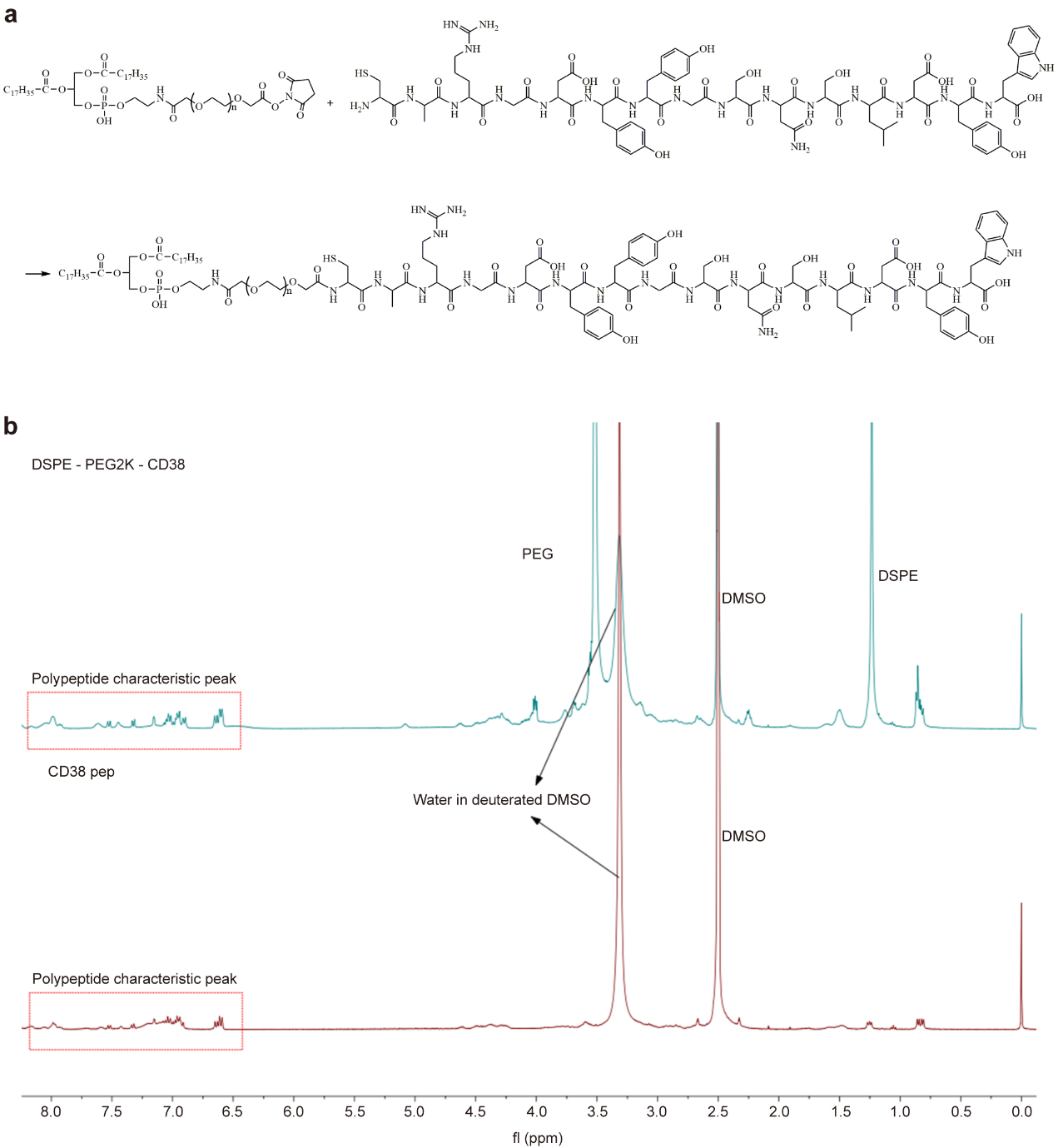


**Fig. S2. Synthesis and characterization of DSPE-PEG-CD38.** (a) The synthetic route for DSPE-PEG-CD38. (b) The DSPE-PEG-CD38 detected by nuclear magnetic hydrogen spectroscopy (1H-NMR).


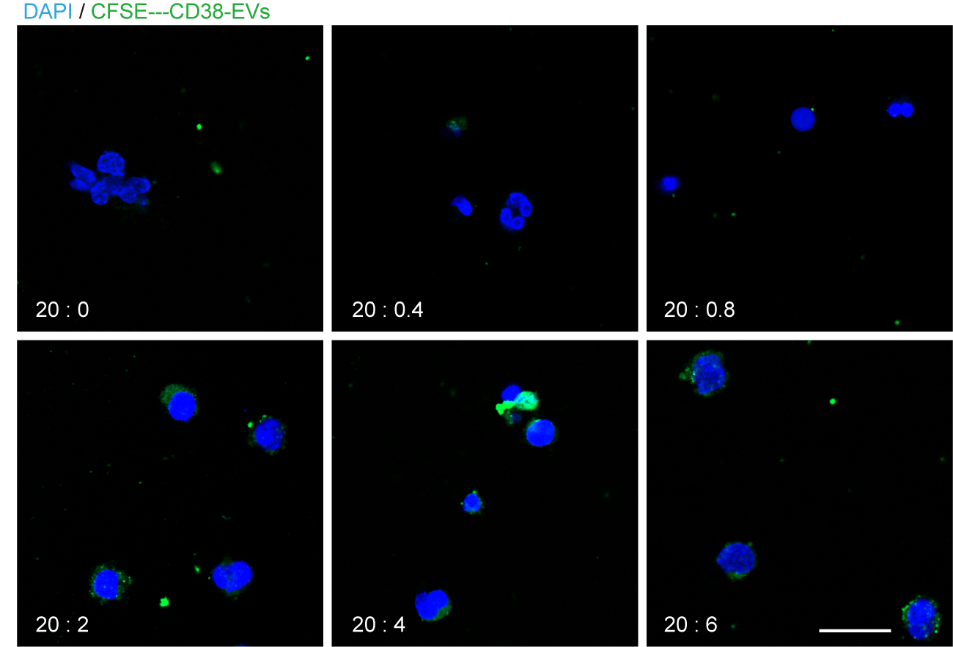


**Fig. S3. Uptake of EVs targeted with varying densities of CD38pep.** CD38-EVs were incubated with RPMI8226 cells in media for 3 hours, and uptake of EVs was observed under laser scanning confocal microscope (*n* = 3) (Scale bars, 50 μm). Incubating concentrations between EVs and CD38pep were measured by EVs protein amount (ug) versus DSPE-PEG-CD38 compound mass (ug).


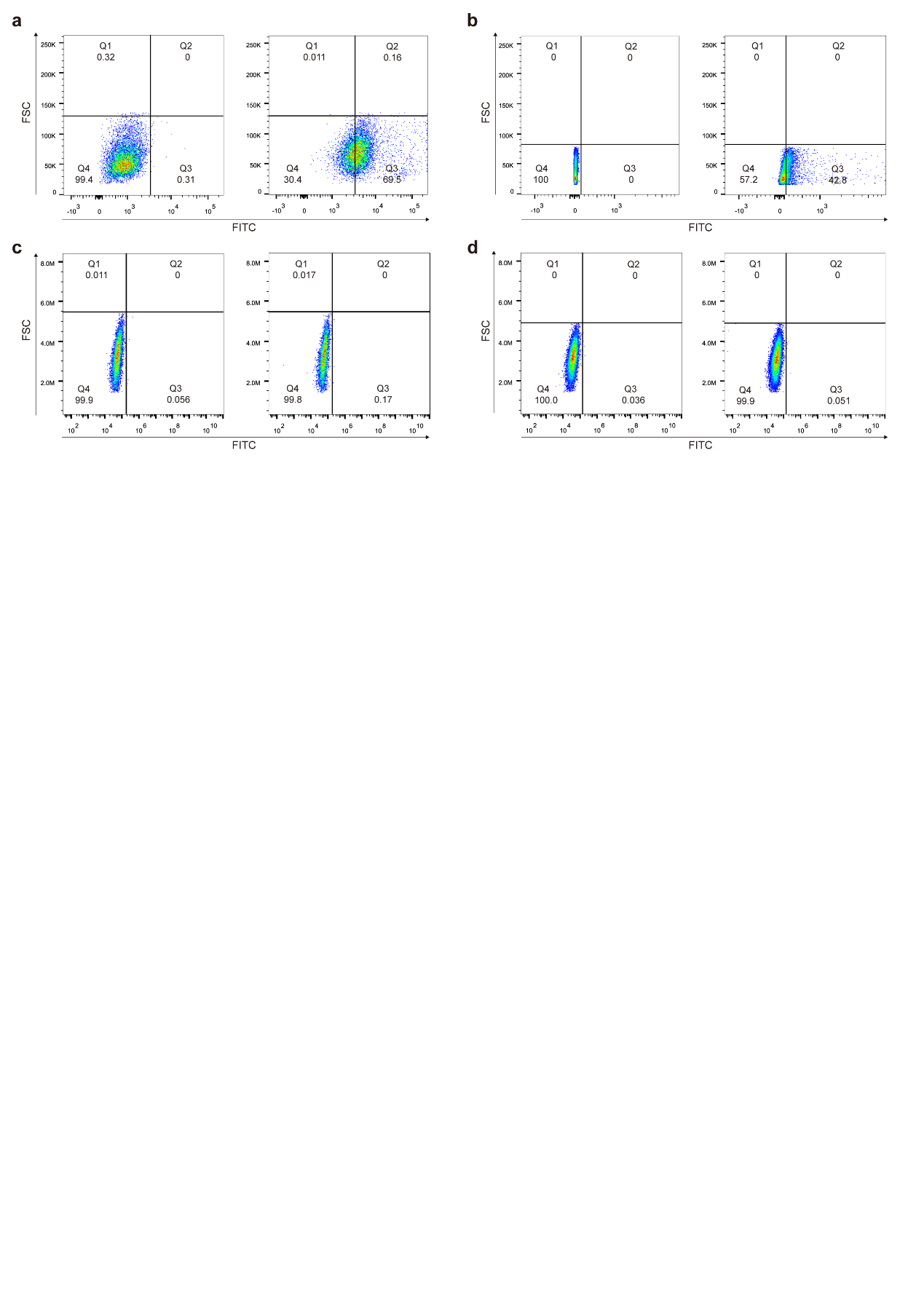


**Fig. S4. CD38 expression in various cell lines.** (a–d) Flow cytometric analysis (FCM) of CD38 expression in RPMI8226 (a), U266 (b), HUVEC (c), and BMSCs (d) after staining with a FITC-conjugated CD38 monoclonal antibody. In each panel, the left plot represents the control group, while the right plot shows the proportion of CD38-positive cells.

**
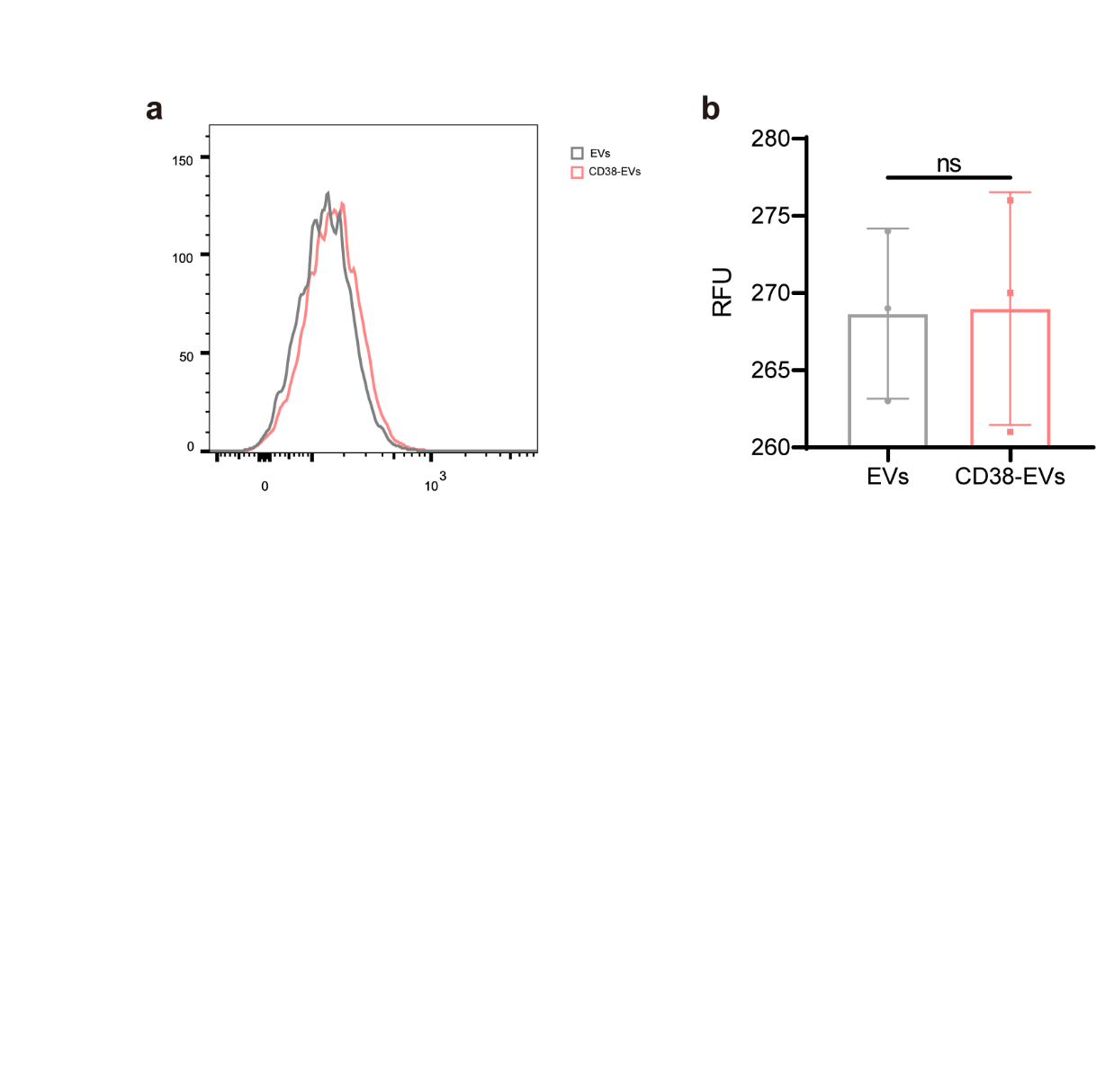
**

**Fig. S5. Targeting capability of CD38-EVs following CD38 antigen blockade.** (a) PKH26-labeled EVs and CD38-EVs were incubated with U266 cells pretreated with daratumumab to block the CD38 antigen for 8 hours, and cell fluorescence was measured by FCM. (b) Kinetics of uptake of EVs and CD38-EVs by U266 cells, measured by RFU. Data are represented as mean ± SD and generated from three independent experiments. Statistical analysis was performed using the one-way ANOVA followed by multiple comparisons with the Tukey post hoc test. *P* value: **p* < 0.05; ***p* < 0.01; ****p* < 0.001; *****p* < 0.0001; ns, nonsignificant.


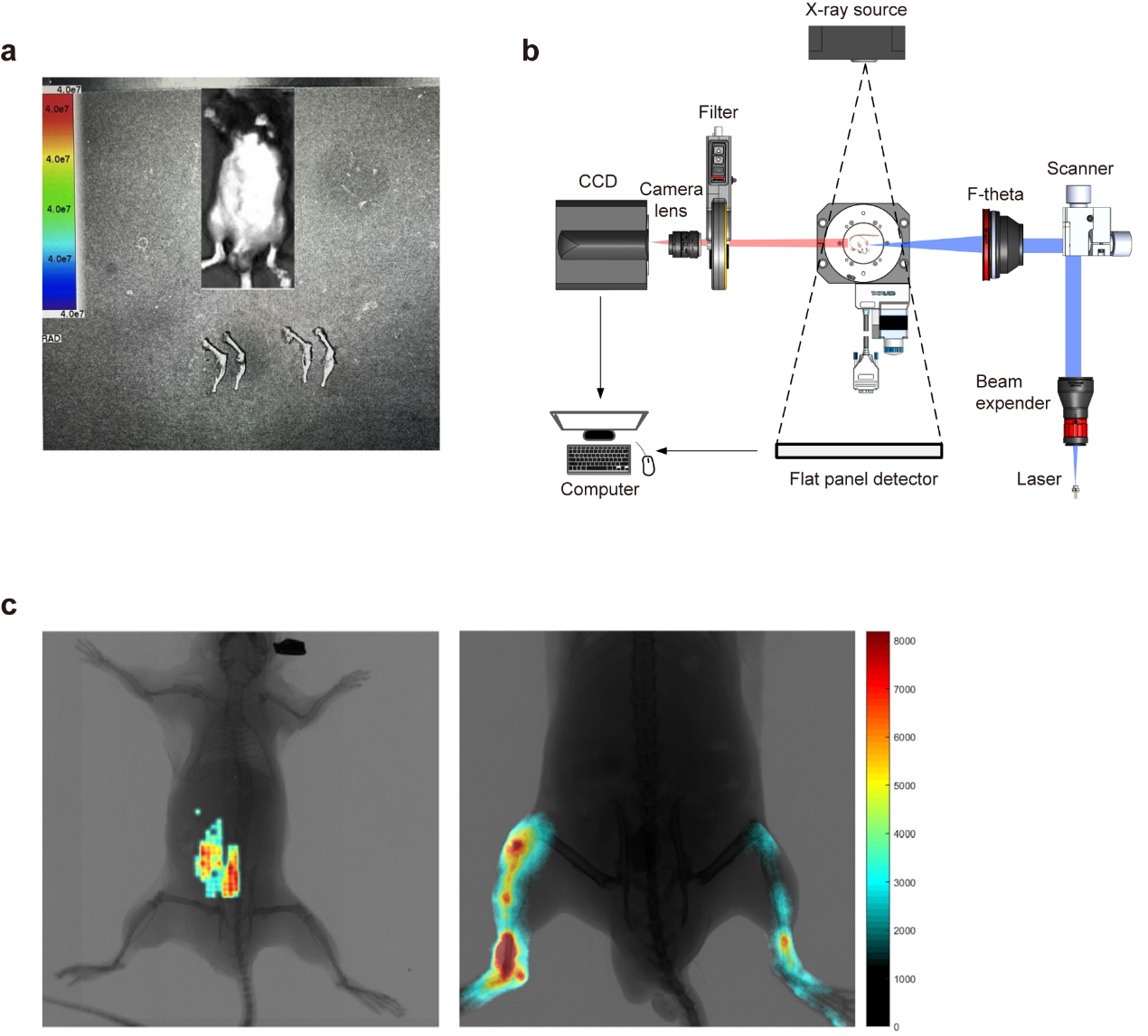


**Fig. S6. FMI fails to detect tiny amounts of EVs in mice and successful construction of the FMP imaging system.** (a) Detection of intravenously injected DiR-labelled EVs (protein quantification, 50 μg) in C57BL/6J using the fluorescent molecular imaging (FMI) of wide-field light, performed 4 hours post-injection. (b) Schematic diagram of a fluorescence molecular projection (FMP) imaging system. (c) Detection of intravenously injected DiR-labelled EVs (protein quantification, 50 μg) in C57BL/6J using FMP, conducted 4 hours post-injection.


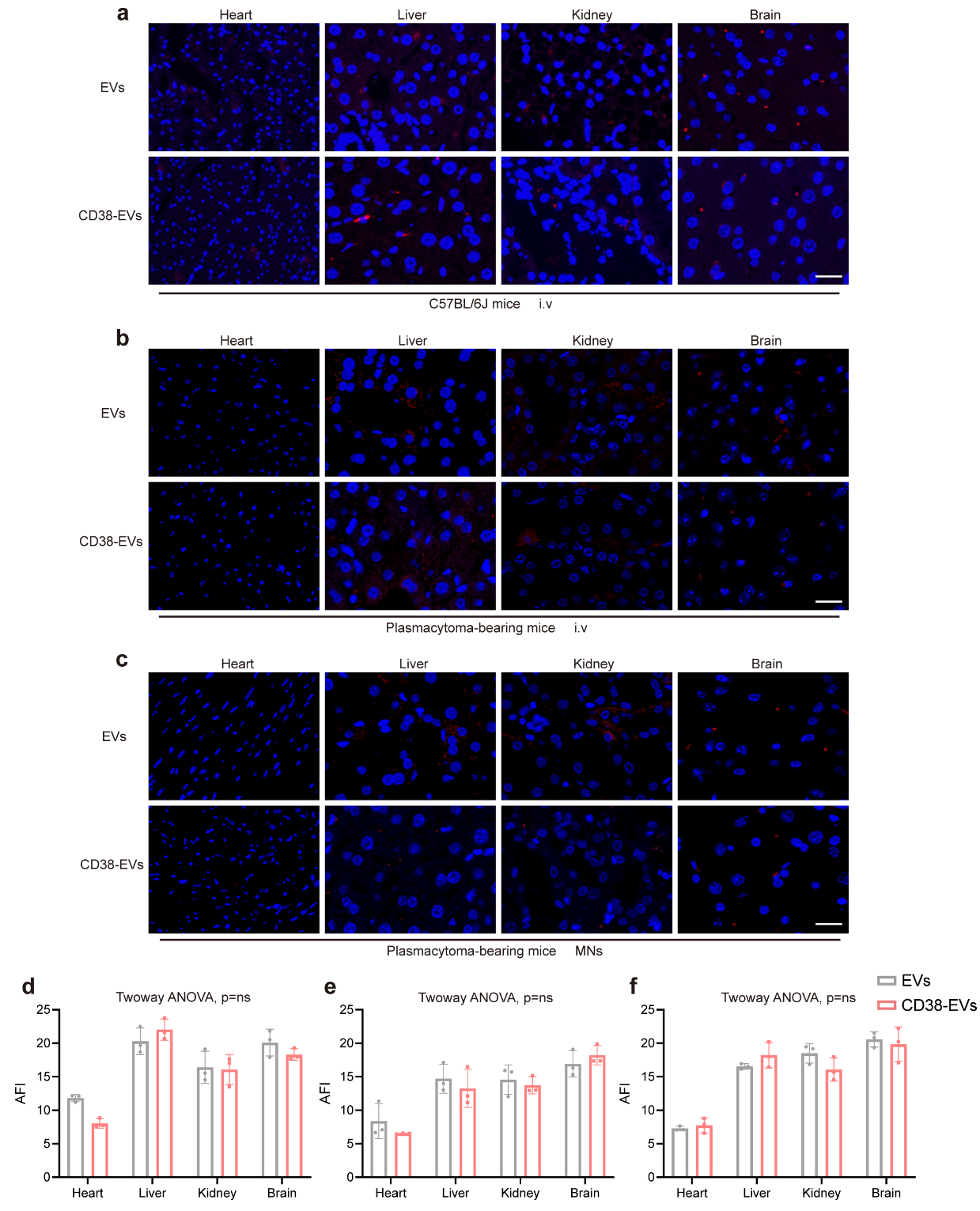


**Fig. S7. Biodistribution of EVs and CD38-EVs in the heart, liver, kidney and brain.** (a, b) Representative fluorescence microscopic images of the distribution of PKH-26-labelled EVs and CD38-EVs in organs of C57BL/6J (a) and EMP mice (b) via intravenous injection at 3 hours (Scale bars, 20 μm). (c) Representative fluorescence microscopic images of the distribution of PKH-26-labelled EVs and CD38-EVs in organs of EMP mice via MNs at 3 hours (Scale bars, 20 μm). (d-f) Quantification of the AFI in the tissue sections in panels (a), (b) and (c) respectively (*n* = 3).


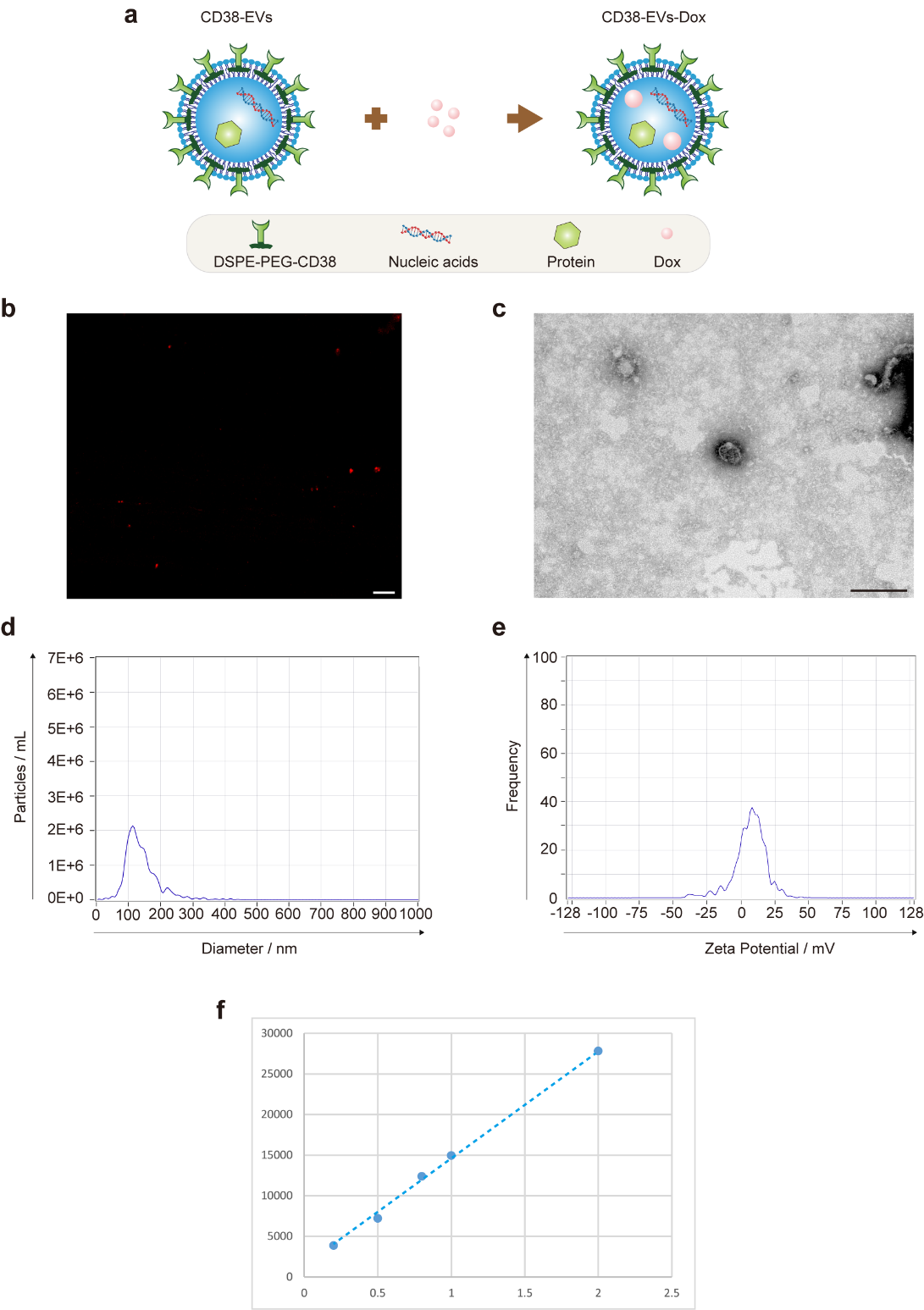


**Fig. S8. Construction and characterization of CD38-EVs-Dox.** (a) Schematic illustration of the construction of CD38-EVs-Dox. (b) CD38-EVs-Dox observed by LSCM (Scale bars, 20 μm). (c) Representative images of CD38-EVs-Dox observed by TEM (Scale bars, 200 nm). (d) Representative images of the size distribution of CD38-EVs-Dox by NTA. (e) Zeta potential of CD38-EVs-Dox determined using a ZetaView analyzer. (f) Standard curve of the doxorubicin concentration determined by liquid chromatography-tandem mass spectrometry (LC-MS/MS).


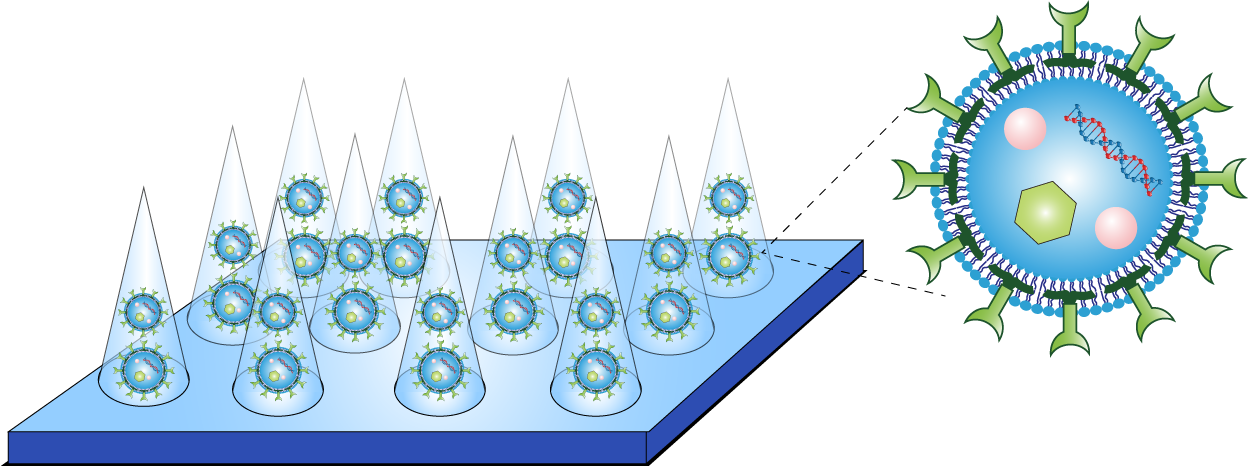


**Fig. S9. Schematic illustration of MNs loaded with CD38-EVs-Dox.**

**
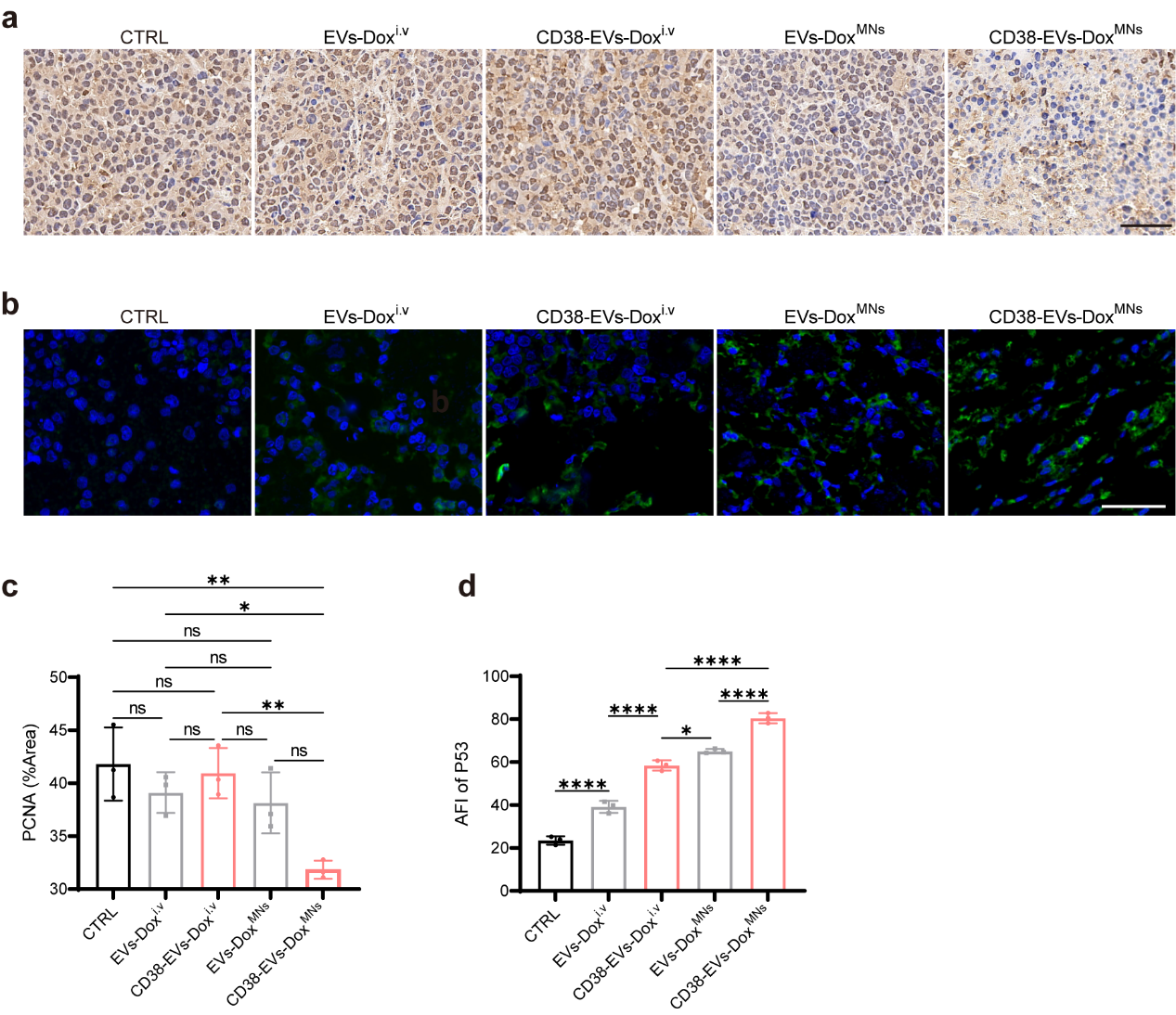
**

**Fig. S10. Antitumor effect of different treatment groups *in vivo*.** (a) Representative images of PCNA IHC in tumor tissues in various groups (Scale bars, 50 μm). (b) Representative images of P53 IF in tumor tissues in various groups (Scale bars, 50 μm). (c) Statistical analysis of PCNA density presented as positive area/total area (*n* = 3). (d) Quantification of the average fluorescence intensity of P53 in tumor sections (n = 3). Data are represented as mean ± SD and statistical analysis was performed using the one-way ANOVA followed by multiple comparisons with the Tukey post hoc test. *P* value: **p* < 0.05; ***p* < 0.01; *****p* < 0.0001; ns, nonsignificant.

**
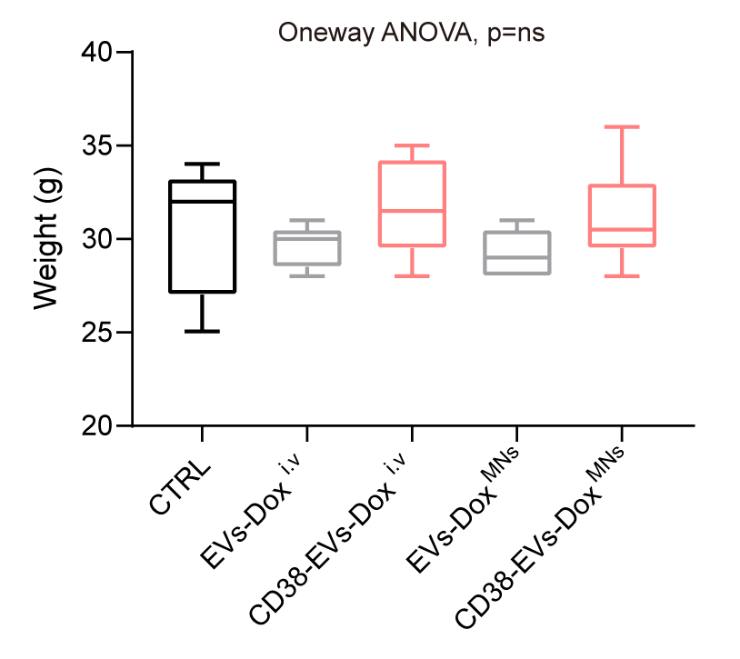
**

**Fig. S11. Statistical analysis of body weights in all treatment groups at 42 days.** The body weights of the mice in the different treatment groups were measured weekly. On the 42^nd^ day, the body weights of all treatment groups were measured for statistical analysis. Each bar represents the mean ± SD of 5-10 mice. Statistical analysis was performed using the one-way ANOVA test, *p* = ns.

**Table. S1.** **Quantification of EVs extracted from umbilical cord MSCs culture medium by high-speed centrifugation.**

| BCA  (μg/ml) * | Mean ± SD  (μg/ml) | NTA  (particles/ml) * | Mean ± SD  (particles/ml) |
| --- | --- | --- | --- |
| 0.23 | 0.27 ± 0.04 | 1.51E+9 | (1.59 ± 0.16)  E+9 |
| 0.28 |  | 1.79E+9 |  |
| 0.31 |  | 1.49E+9 |  |

*: μg/ml represents the mass of EV protein (μg) extracted from 1 ml of umbilical cord MSC-conditioned medium, while particles/ml denotes the number of EV particles extracted from 1 ml of umbilical cord MSC-conditioned medium.

**Table. S2.** **Determination of doxorubicin loading capacity of CD38-EVs.**

| Peak area | Dox  (μg/mL) (W_0_) | CD38-EVs-Dox  (μg/mL) (W_1_) | Loading rate  (%) | Mean ± SD  (%) |
| --- | --- | --- | --- | --- |
| 15026.00 | 1.03 | 5.00 | 20.61 | 21.88 ± 1.16 |
| 16543.00 | 1.14 | 5.00 | 22.86 |  |
| 16087.00 | 1.11 | 5.00 | 22.18 |  |

Dox, doxorubicin; SD, standard deviation. Drug loading rate = W0/W1×100%. W0 is the mass of the drug-loaded, and W1 is the total mass of the drug-loaded CD38-EVs.
