# Supplementary material for: Targeting capacity, safety and efficacy of engineered extracellular vesicles delivered by transdermal microneedles to treat plasmacytoma in mice
Source: Clin Transl Med. 2025 May 2;15(5):e70327. doi: 10.1002/ctm2.70327 (PMC12048306; doi:10.1002/ctm2.70327)
Supplement: Supplementary file 2 — Supporting Information [file CTM2-15-e70327-s001.docx]

**Table. S1.** **Quantification of EVs extracted from umbilical cord MSCs culture medium by high-speed centrifugation.**

| BCA  (μg/ml) * | Mean ± SD  (μg/ml) | NTA  (particles/ml) * | Mean ± SD  (particles/ml) |
| --- | --- | --- | --- |
| 0.23 | 0.27 ± 0.04 | 1.51E+9 | (1.59 ± 0.16)  E+9 |
| 0.28 |  | 1.79E+9 |  |
| 0.31 |  | 1.49E+9 |  |

*: μg/ml represents the mass of EV protein (μg) extracted from 1 ml of umbilical cord MSC-conditioned medium, while particles/ml denotes the number of EV particles extracted from 1 ml of umbilical cord MSC-conditioned medium.

**Table. S2.** **Determination of doxorubicin loading capacity of CD38-EVs.**

| Peak area | Dox  (μg/mL) (W_0_) | CD38-EVs-Dox  (μg/mL) (W_1_) | Loading rate  (%) | Mean ± SD  (%) |
| --- | --- | --- | --- | --- |
| 15026.00 | 1.03 | 5.00 | 20.61 | 21.88 ± 1.16 |
| 16543.00 | 1.14 | 5.00 | 22.86 |  |
| 16087.00 | 1.11 | 5.00 | 22.18 |  |

Dox, doxorubicin; SD, standard deviation. Drug loading rate = W0/W1×100%. W0 is the mass of the drug-loaded, and W1 is the total mass of the drug-loaded CD38-EVs.
