# Supplementary figures and images for: Targeting capacity, safety and efficacy of engineered extracellular vesicles delivered by transdermal microneedles to treat plasmacytoma in mice
Source: Clin Transl Med. 2025 May 2;15(5):e70327. doi: 10.1002/ctm2.70327 (PMC12048306; doi:10.1002/ctm2.70327)

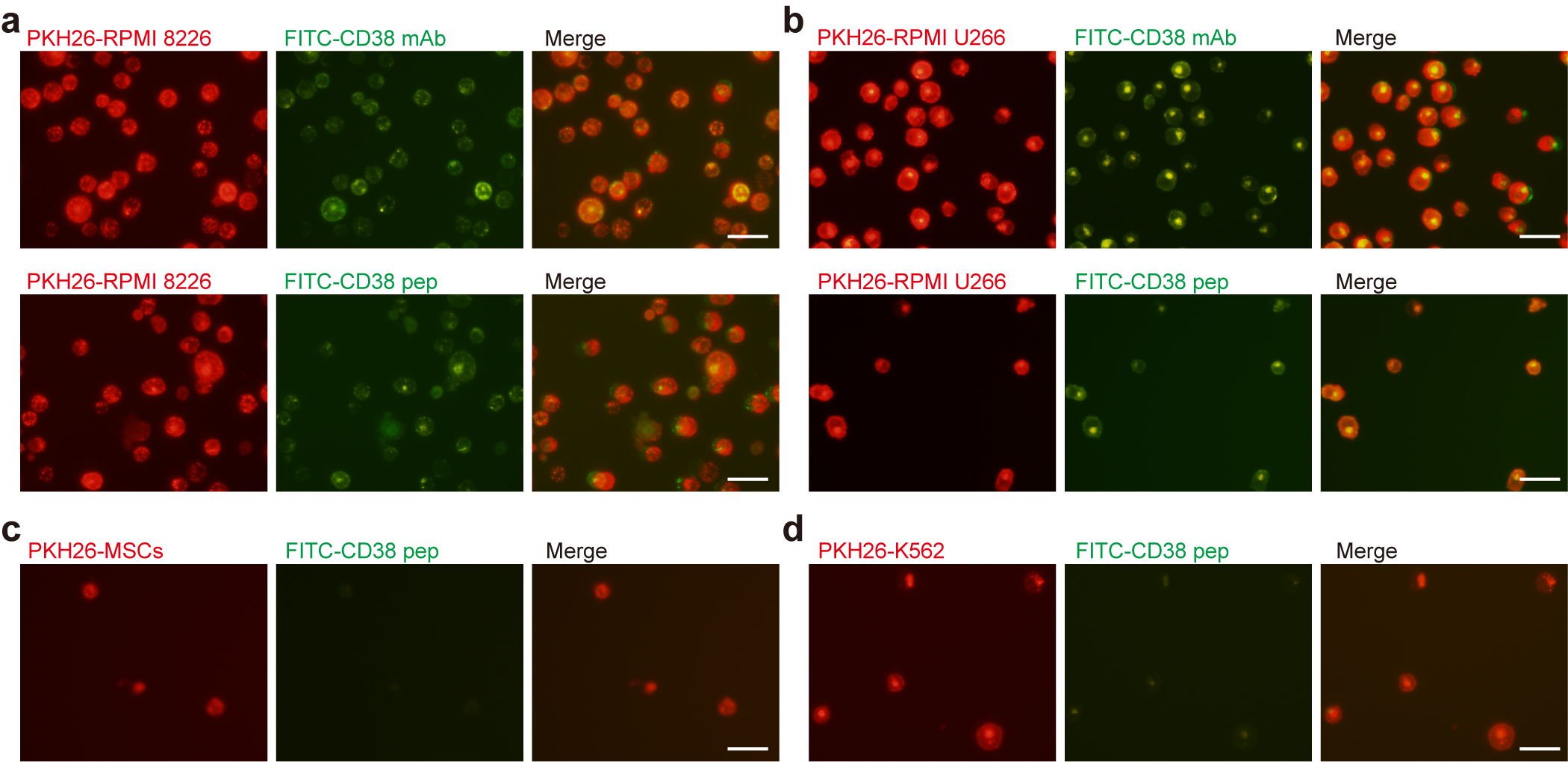

Supplement: Supplementary file 3 — Supporting Information [file CTM2-15-e70327-s007.tif]

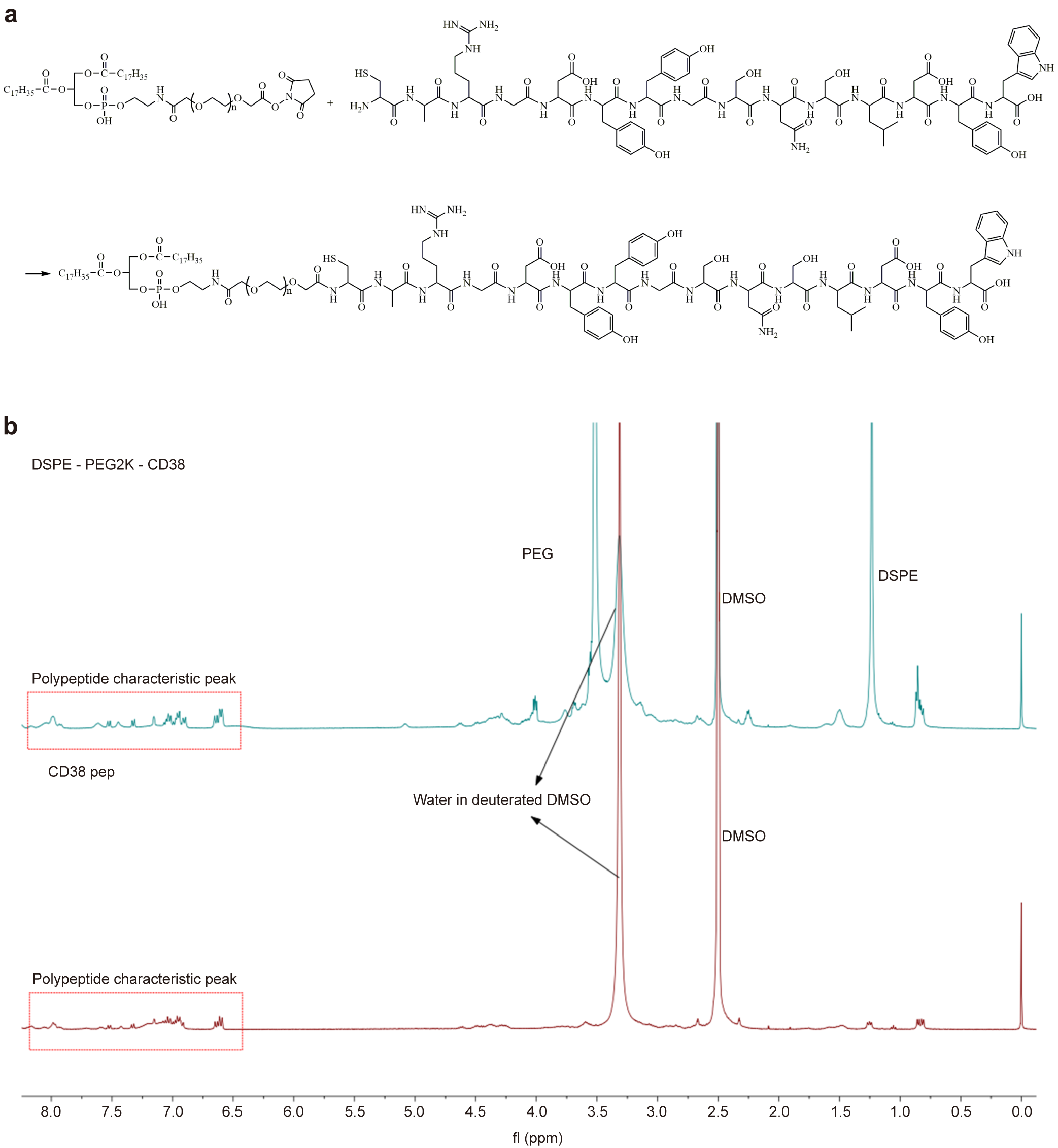

Supplement: Supplementary file 4 — Supporting Information [file CTM2-15-e70327-s008.tif]

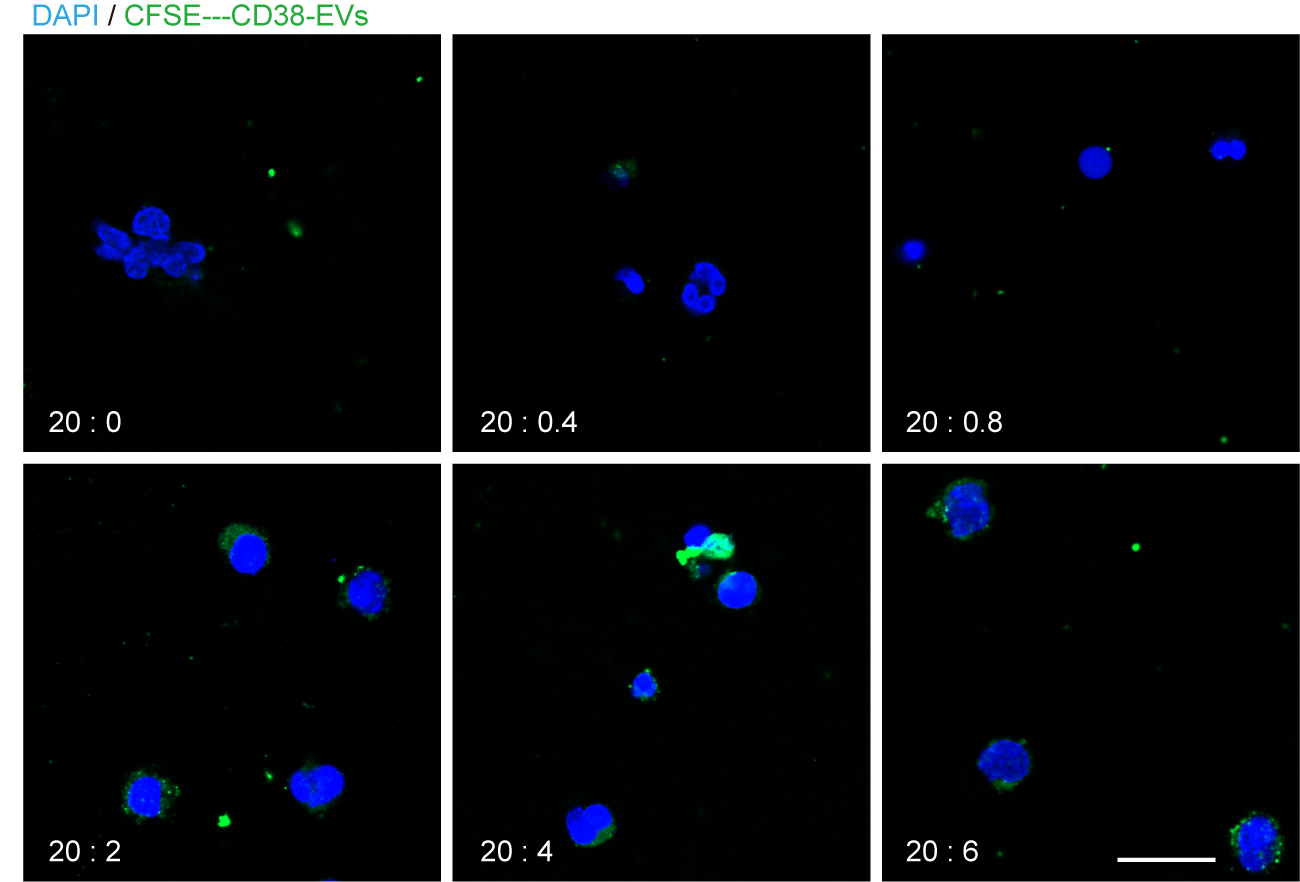

Supplement: Supplementary file 5 — Supporting Information [file CTM2-15-e70327-s002.tif]

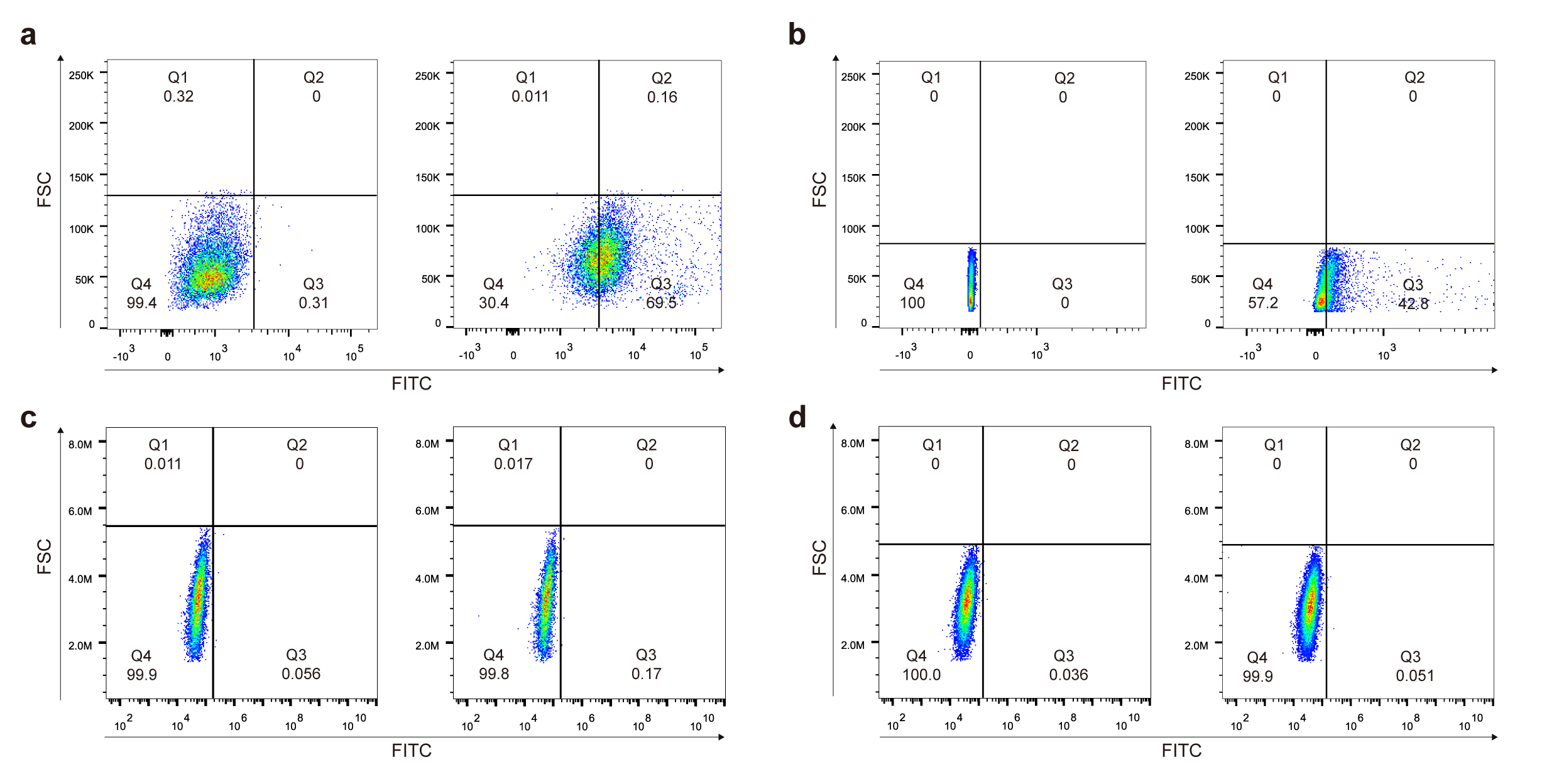

Supplement: Supplementary file 6 — Supporting Information [file CTM2-15-e70327-s011.tif]

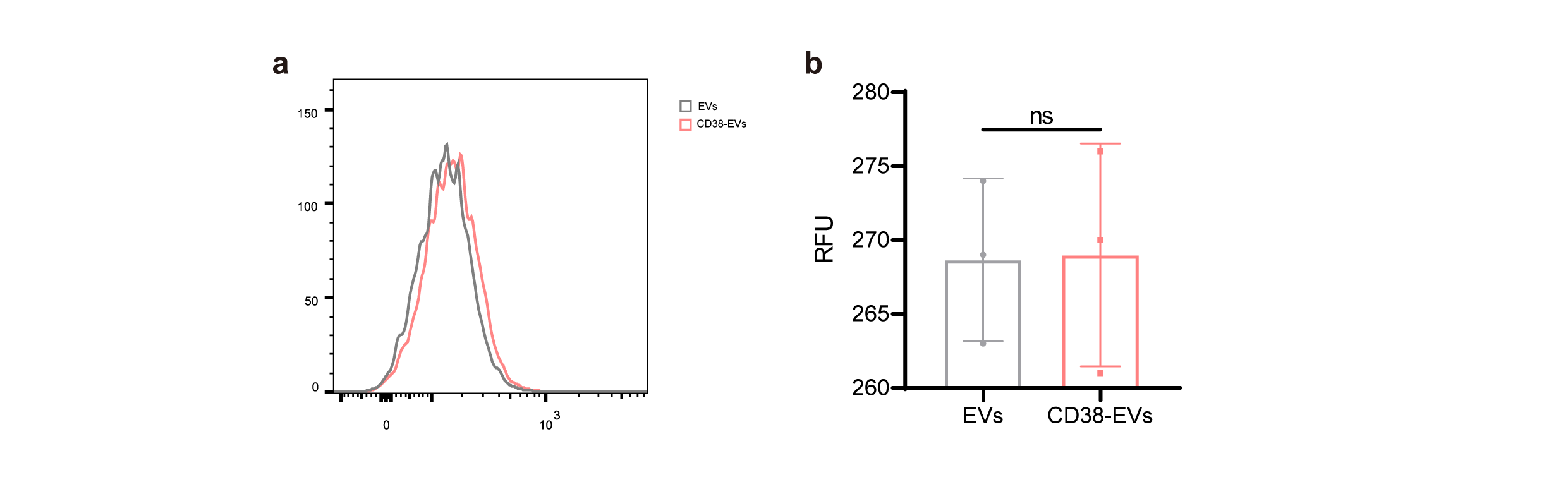

Supplement: Supplementary file 7 — Supporting Information [file CTM2-15-e70327-s006.tif]

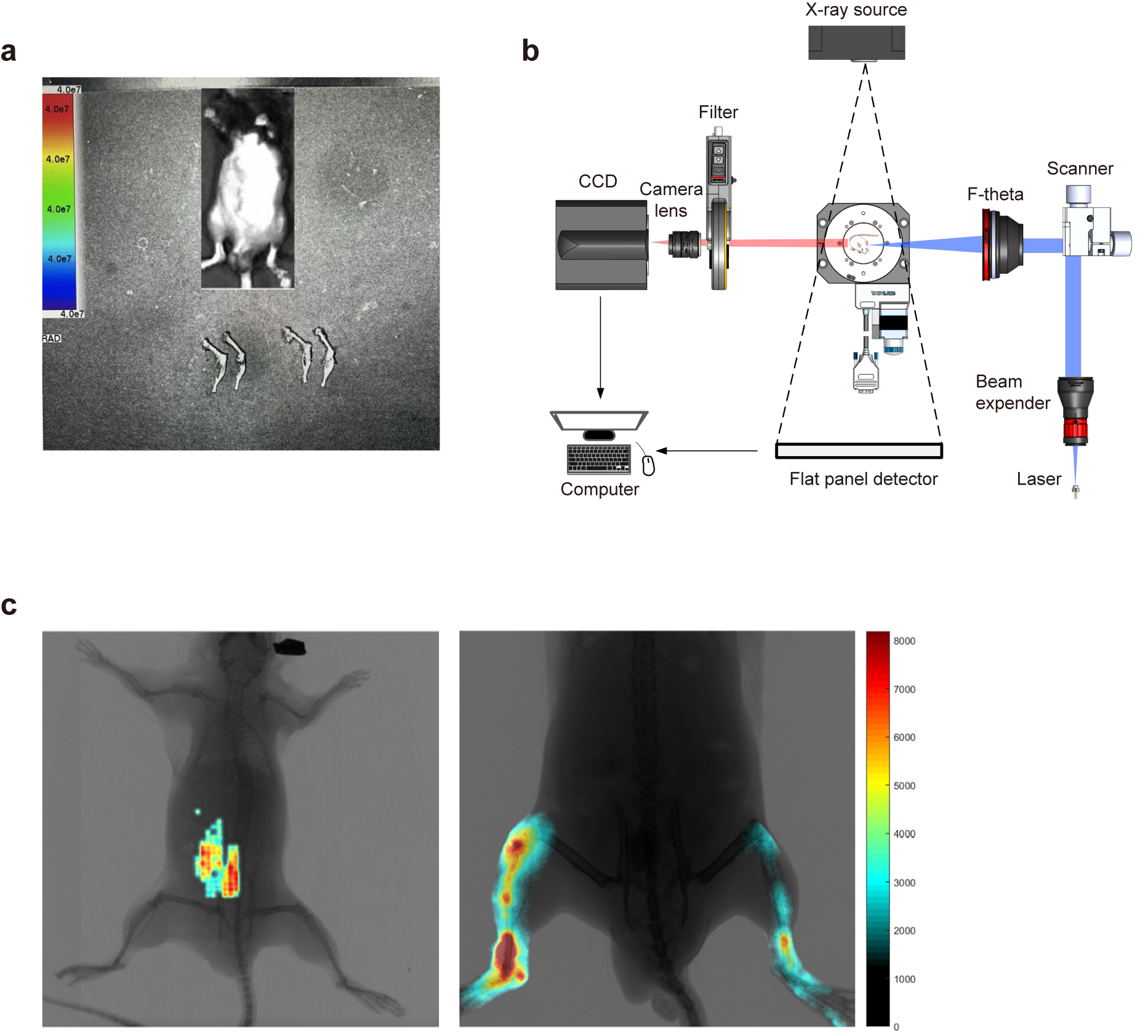

Supplement: Supplementary file 8 — Supporting Information [file CTM2-15-e70327-s012.tif]

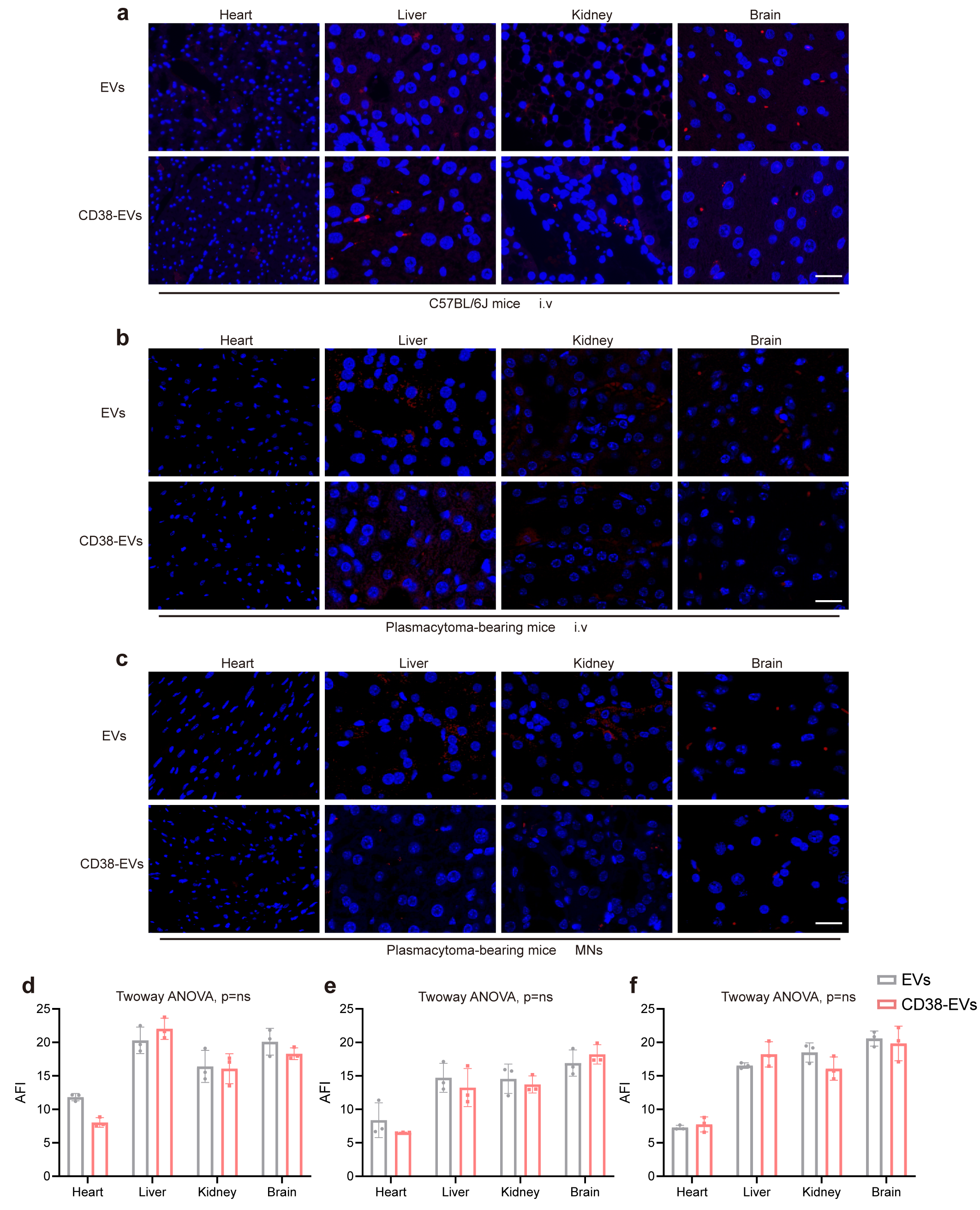

Supplement: Supplementary file 9 — Supporting Information [file CTM2-15-e70327-s009.tif]

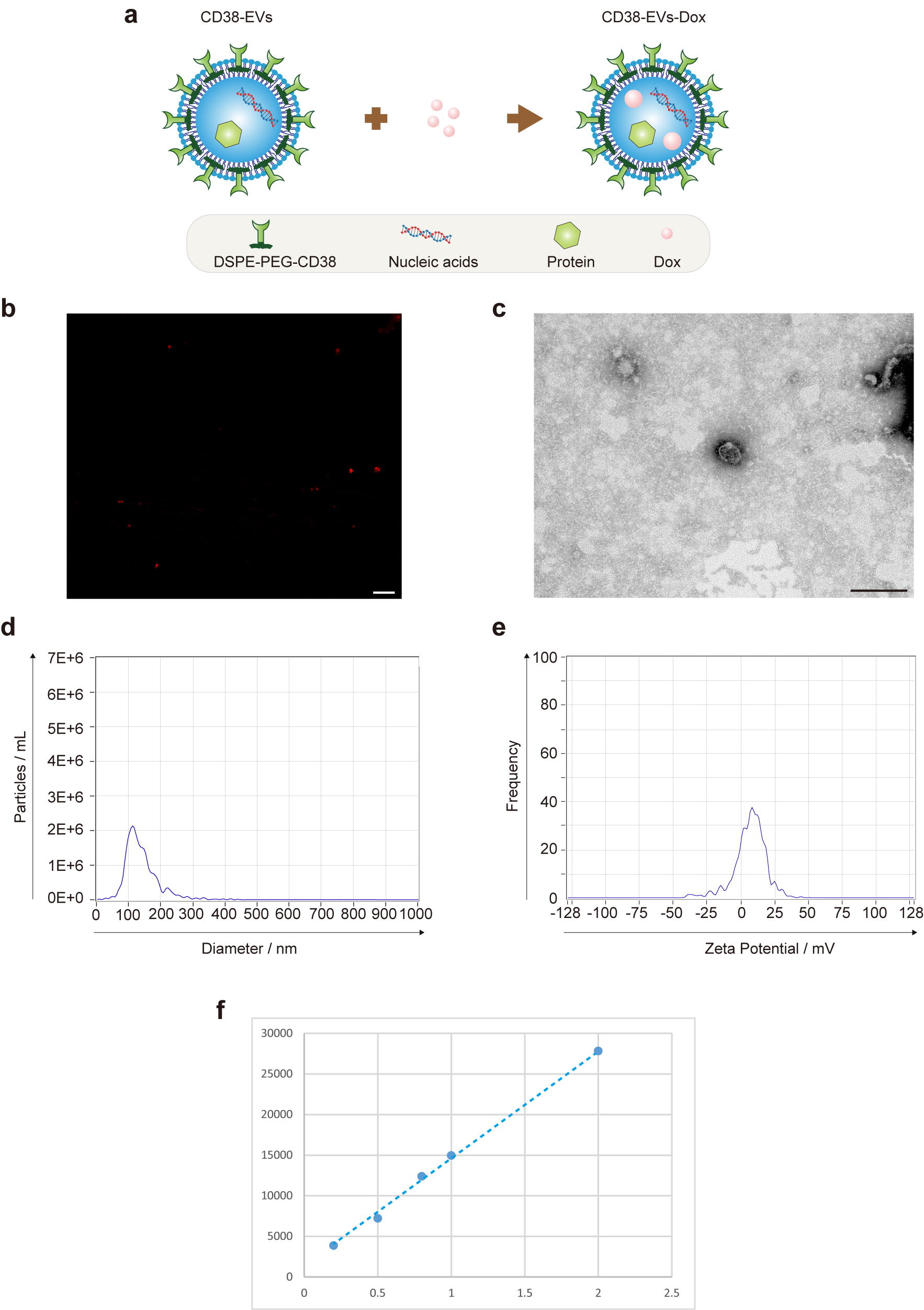

Supplement: Supplementary file 10 — Supporting Information [file CTM2-15-e70327-s013.tif]

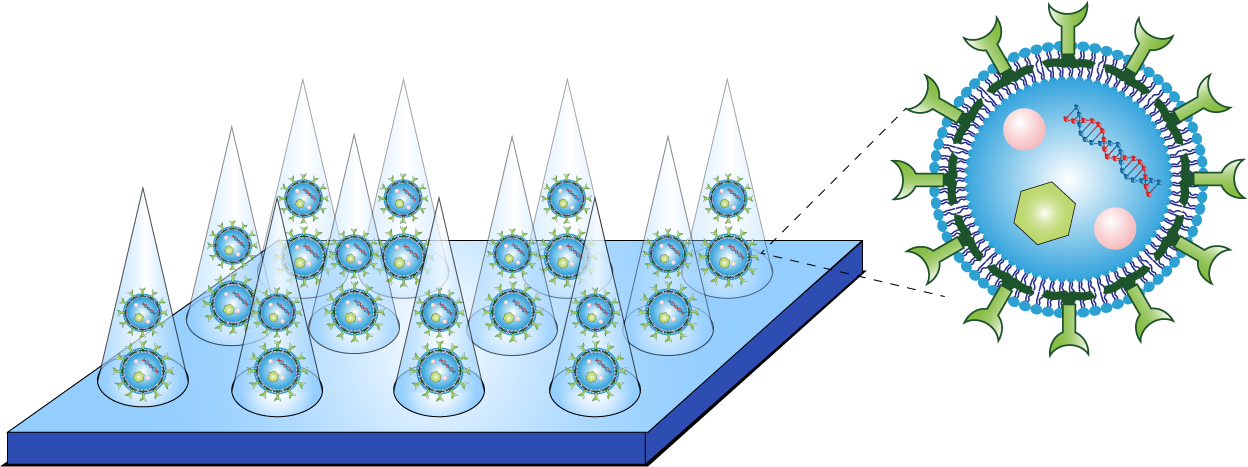

Supplement: Supplementary file 11 — Supporting Information [file CTM2-15-e70327-s004.tif]

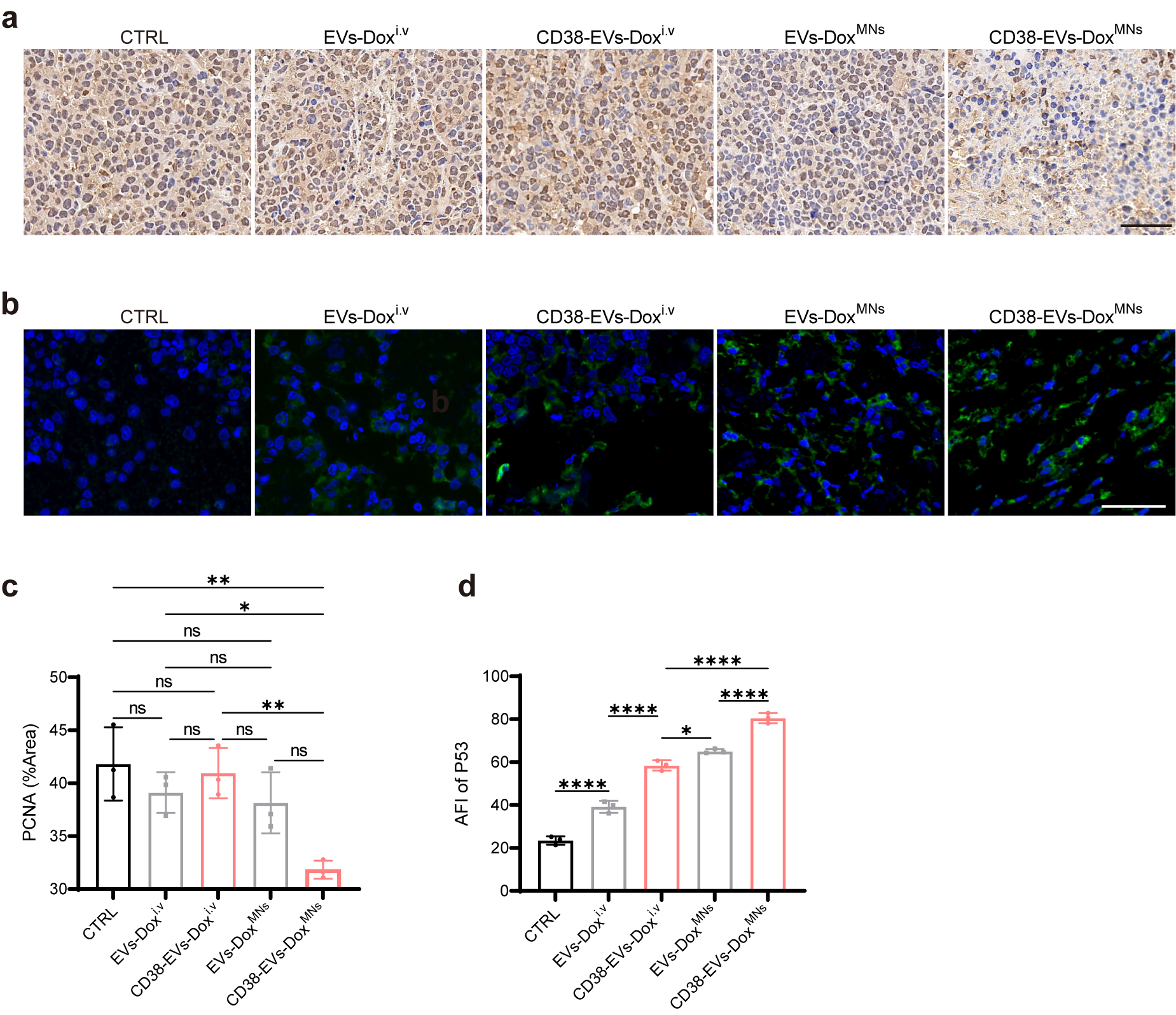

Supplement: Supplementary file 12 — Supporting Information [file CTM2-15-e70327-s010.tif]

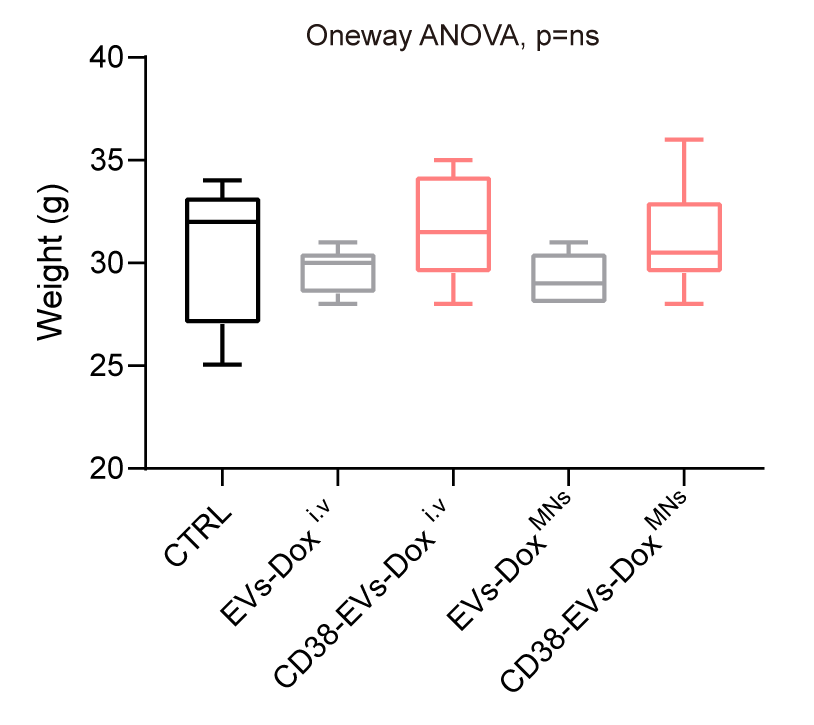

Supplement: Supplementary file 13 — Supporting Information [file CTM2-15-e70327-s003.tif]
